# Time-Dependent Pathological Changes in Hypoperfusion-Induced Abdominal Aortic Aneurysm

**DOI:** 10.3390/biology10020149

**Published:** 2021-02-14

**Authors:** Hirona Kugo, Wanida Sukketsiri, Hiroki Tanaka, Rena Fujishima, Tatsuya Moriyama, Nobuhiro Zaima

**Affiliations:** 1 Department of Applied Biological Chemistry, Graduate School of Agriculture, Kindai University, 204-3327 Nakamachi, Nara 631-8505, Japan; hirona.kugo@gmail.com (H.K.); rena21fujishima@gmail.com (R.F.); tmoriyama@nara.kindai.ac.jp (T.M.); 2 Department of Pharmacology, Faculty of Science, Prince of Songkla University, Songkhla 90110, Thailand; wanida.su@psu.ac.th; 3 Department of Medical Physiology, Hamamatsu University School of Medicine, Hamamatsu 431-3192, Japan; 44htanaka@gmail.com; 4 Agricultural Technology and Innovation Research Institute, Kindai University, Nara 631-8505, Japan

**Keywords:** abdominal aortic aneurysm, hypoperfusion, vascular disease, hypoxia, inflammation

## Abstract

**Simple Summary:**

Abdominal aortic aneurysm (AAA) is a vascular disease that involves gradual dilation of the abdominal aorta and has a high mortality due to rupture. Hypoperfusion due to the obstruction of vasa vasorum, which is a blood supply system in the aortic wall, may be an important factor involved in AAA pathophysiology. A time-dependent analysis is important to understand the pathological cascade following hypoperfusion in the aortic wall. In our study, time-dependent analysis using a hypoperfusion-induced animal model showed that the dynamics of many AAA-related factors might be associated with the increased hypoxia-inducible factor-1α level. Hypoperfusion due to stenosis of the vasa vasorum might be a new drug target for AAA therapeutics.

**Abstract:**

Hypoperfusion due to vasa vasorum stenosis can cause wall hypoxia and abdominal aortic aneurysm (AAA) development. Even though hypoperfusion is an important contributor toward pathological changes in AAA, the correlation between hypoperfusion and AAA is not fully understood. In this study, a time-dependent semi-quantitative pathological analysis of hypoperfusion-induced aortic wall changes was performed to understand the mechanisms underlying the gradual degradation of the aortic wall leading to AAA formation. AAA-related factors evaluated in this study were grouped according to the timing of dynamic change, and five groups were formed as follows: first group: angiotensin II type 1 receptor, endothelin-1 (ET-1), and malondialdehyde (MDA); second group: matrix metalloproteinase (MMP)-2, -9, -12, M1 macrophages (Mac387+ cells), and monocyte chemotactic protein-1; third group: synthetic smooth muscle cells (SMCs); fourth group: neutrophil elastase, contractile SMCs, and angiotensinogen; and the fifth group: M2 macrophages (CD163+ cells). Hypoxia-inducible factor-1α, ET-1, MDA, and MMP-9 were colocalized with alpha-smooth muscle actin cells in 3 h, suggesting that hypoperfusion-induced hypoxia directly affects the activities of contractile SMCs in the initial stage of AAA. Time-dependent pathological analysis clarified the cascade of AAA-related factors. These findings provide clues for understanding complicated multistage pathologies in AAA.

## 1. Introduction

Abdominal aortic aneurysm (AAA) is a vascular disease characterized by progressive dilation of the abdominal aorta. The risk factors for AAA include smoking, hypertension, male sex, and older age [1]. The risk of AAA rupture increases with an increase in AAA diameter [2]. The increased AAA diameter is caused by the continuous breakdown of the aortic wall structure, which is associated with several AAA-related factors [3]. The exact mechanisms underlying the breakdown of the aortic wall structure are not entirely clear.

The normal aortic wall consists of the intima, media, and adventitia. In the adventitia, small blood vessels called the vasa vasorum (VV) oxygenate the aortic wall [4]. Abnormal diffusion of oxygen to the aortic wall causes local hypoxia, which is associated with vascular remodeling [5,6]. In a previous study, we reported that VV stenosis causes hypoperfusion of the human abdominal aortic wall [7]. Similarly, induction of hypoperfusion in the aortic wall causes AAA formation and rupture in rats [8,9], and the pathologies of hypoperfusion-induced animal AAA are consistent with those of human AAA. Similar to human AAA, aortic wall dilation [10], aneurysm rupture [10], adipogenesis in the adventitial wall [11,12,13], the presence of intraluminal thrombus [6,14], vascular wall thickness [15], medial wall thinning with smooth muscle cell (SMC) depletion [16], degradation of collagen and elastin fibers [10], gelatinolytic activity [17,18], oxidative stress due to the increased production of reactive oxygen species (ROS) [19,20], and VV stenosis [7] have been reported in hypoperfusion-induced AAA animal models [8,9,21]. Levels of hypoxia-inducible factor-1α (HIF-1α) [22], matrix metalloproteinase (MMP)-2 [17], MMP-9 [17], MMP-12 [23], M1 [24] and M2 [25] macrophages, and monocyte chemotactic protein-1 (MCP-1) [26], which are increased in the human abdominal aortic wall, were also significantly increased in the aneurysmal wall (the dilated region) of the hypoperfusion-induced AAA animal model compared with the non-dilated normal region. Alpha-smooth muscle actin (α-SMA)+ cells (contractile SMCs) in the medial wall, which are decreased in human AAA [27], were significantly decreased in the hypoperfusion-induced aortic wall. These reports suggest that hypoperfusion in the aortic wall may be an important factor in AAA formation. However, the pathological cascade following hypoperfusion remains largely unknown. Time-dependent pathological analysis is effective for identifying hypoperfusion-induced factors that can be closely associated with AAA development. In this study, we evaluated the time-dependent pathological changes in hypoperfusion-induced walls to clarify the mechanisms underlying hypoperfusion-induced AAA formation.

## 2. Materials and Methods

### 2.1. Animals

All animal experiments were approved by the Kindai University Animal Care and Use Committee and performed according to the Kindai University Animal Experimentation Regulations (approval number: KAAG-31-006). Six-week-old male Sprague-Dawley rats (Japan SLC, Inc., Shizuoka, Japan) were maintained in a room at 25 ± 1 °C with a 12-h light and 12-h dark cycle with free access to food and water. To collect aortic tissue and evaluate the pathology at several points from the induction of hypoperfusion to 28 days later, rats were divided into day 0 (n = 7), day 2 (n = 6), day 3 (n = 5), day 5 (n = 6), day 7 (n = 6), day 10 (n = 5), day 14 (n = 6), day 21 (n = 14), and day 28 (n = 7) groups. In the experiment to evaluate aortic pathology within a short period, rats were divided to 0 h (n = 6), 3 h (n = 7), 6 h (n = 6), and 24 h (n = 7) groups. After a 1-week habituation period, the abdominal aorta was subjected to hypoperfusion by ligation of the aortic wall to induce AAA. After the induction of hypoperfusion, the animal was sacrificed and the abdominal aorta was collected after 2, 3, 5, 7, 10, 14, 21, and 28 days according to the assigned group. In the experiment within a short period, the animal was sacrificed and the abdominal aorta was collected after 3, 6, and 24 h after treatment. In the day 0 and 0 h groups, the abdominal aorta in which hypoperfusion was not induced was collected. All surgeries were performed under anesthesia with minimal suffering.

### 2.2. Induction of Hypoperfusion in the Abdominal Aortic Wall

Hypoperfusion in the aortic wall was induced as described in previous studies [9,21,28]. The infrarenal aorta was exfoliated from the perivascular adipose tissue (Figure S1A), and vessels branching were ligated with 5–0 silk sutures (Akiyama Seisakusyo Co., Tokyo, Japan) to block the blood supply (Figure S1B). To block aortic blood flow, the aorta was ligated just below the renal artery and just above the bifurcation of the aorta (Figure S1C). A small incision was created by cutting the aortic wall (Figure S1D), and a polyurethane catheter (Medikit, Tokyo, Japan) shortened to 9 mm in length, was inserted (Figure S1E). The incision was repaired with a 6–0 monofilament suture (Alfresa Pharma, Osaka, Japan) (Figure S1F). The aortic wall was ligated over the inserted catheter using a 5–0 silk suture (Figure S1G). The 5-0 silk suture to block the blood in the aorta was removed to restore the blood flow (Figure S1H).

### 2.3. Histological Analysis

The diameter of the abdominal aorta was measured using digital calipers (A&D, Tokyo, Japan). Isolated tissues were fixed in 4% paraformaldehyde (PFA) (Nacalai Tesque, Kyoto, Japan), soaked in sucrose (10%, 15%, and 20%), and embedded in O.C.T. compound (Sakura Finetek Japan Co., Ltd., Tokyo, Japan). These tissue samples were then stored at −80 °C until use.

Isolated aorta cross-sections (10 µm thick) were prepared using a cryostat (CM1850; Leica Microsystems, Wetzlar, Germany) and mounted on glass slides. Aortic walls were visualized with hematoxylin-eosin, picrosirius red, Elastica van Gieson, and immunohistochemical staining. Immunohistochemical staining was performed as described in previous studies [6,18,25] using antibodies for the following proteins: rabbit anti-MMP-2 (1:50; Thermo Scientific, San Jose, CA, USA), goat anti-MMP-9 (1:50; Santa Cruz Biotechnology, Dallas, TX, USA), rabbit anti-MMP-12 (1:100; Bioss Antibodies, Woburn, MA, USA), rabbit anti-neutrophil elastase (1:100; Bioss Antibodies), mouse anti-monocytes/macrophages (Mac387) (1:50; Bio-Rad Laboratories, Hercules, CA, USA), rabbit anti-CD163 (1:100; Bioss Antibodies), rabbit anti- MCP-1 (1:50; Bioss Antibodies), mouse anti-α-SMA (1:400; Santa Cruz Biotechnology), mouse anti-non-muscle myosin heavy chain (SMemb) (1:100; Yamasa, Chiba, Japan), mouse anti-angiotensinogen (1:50; Novus Biologicals, Littleton, CO, USA), rabbit anti-angiotensin II (AngII) type 1 (AT1) receptor (1:100; Advanced Targeting Systems, San Diego, CA, USA), rabbit anti-endothelin-1 (ET-1) (1:100; Abcam, Tokyo, Japan), and rabbit anti-malondialdehyde (MDA) (1:100; Abcam). Quantitative analyses of the immunohistochemical staining were performed using ImageJ software (National Institutes of Health, Bethesda, MD, USA). The area of positive staining in immunohistochemistry was calculated by binarizing the image into black and white using ImageJ. Sections of the negative control were not subjected to the primary antibodies.

### 2.4. Microscopy

Aortic wall sections were observed under an optical microscope (CX23, Olympus Corporation, Tokyo, Japan) with a ×40 objective, connected to a camera (E-620, Olympus Corporation, Tokyo, Japan) with a 4032 × 3024 pixel resolution. Immunofluorescence staining was evaluated using a fluorescence microscope (ECLIPSE E200, Nikon Corporation, Tokyo, Japan) with a ×10 objective, connected to Basler PowerPack for microscopy with a microscopy pulse (Basler AG, Ahrensburg, Germany). Basler microscopy software (Version 1.1, Basler AG) was used for image acquisition. The microscope was equipped with a fluorescence illumination system (E2-FM, Nikon Corporation, Tokyo, Japan) with a DAPI filter, FITC filter, and Texas red filter.

### 2.5. Statistical Analyses

Values are expressed as mean ± SEM. Statistically significant differences were determined using the steel test and Mann-Whitney U test. Statistical significance was set at p < 0.05. Statistical analyses were performed using StatView software (version 5.0; SAS Institute, Cary, NC, USA) and EZR software [29].

## 3. Results

### 3.1. Protein Levels in AAA-Related Factors in the Hypoperfusion-Induced Animal Model

**Figure 1:**
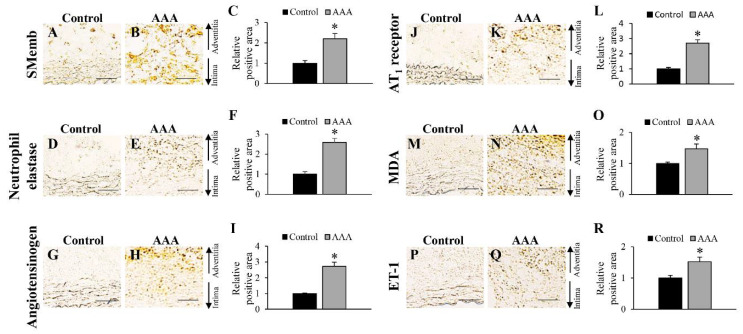
Positive areas for synthetic vascular smooth muscle (SMemb), neutrophil elastase, angiotensinogen, angiotensin II type 1 (AT_1_) receptor, malondialdehyde (MDA), and endothelin-1 (ET-1) on 28 days after the induction of hypoperfusion. (**A**,**B**) Representative images of the immunostaining for SMemb (scale bar = 50 µm). (**C**) Quantification of areas positive for SMemb in the vascular wall. (**D**,**E**) Representative images of the immunostaining for neutrophil elastase (scale bar = 50 µm). (**F**) Quantification of areas positive for neutrophil elastase in the vascular wall. (**G**,**H**) Representative images of the immunostaining for angiotensinogen (scale bar = 50 µm). (**I**) Quantification of areas positive for angiotensinogen in the vascular wall. (**J**,**K**) Representative images of the immunostaining for AT_1_ receptor (scale bar = 50 µm). (**L**) Quantification of areas positive for AT_1_ receptor in the vascular wall. (**M**,**N**) Representative images of the immunostaining for MDA (scale bar = 50 µm). (**O**) Quantification of areas positive for MDA in the vascular wall. (**P**,**Q**) Representative images of the immunostaining for ET-1 (scale bar = 50 µm). (**R**) Quantification of areas positive for ET-1 in the vascular wall. Data are expressed as the mean ± SEM. * *P* < 0.05 versus control wall. SMemb and neutrophil elastase: control wall (n = 9) and AAA wall (n = 10), AGT and AT_1_ receptor: control wall (n = 8) and AAA wall (n = 10), MDA: control wall (n = 10) and AAA wall (n = 12), and ET-1: control wall (n = 9) and AAA wall (n = 14).

Before the time-dependent pathological analysis of AAA wall formation in the hypoperfusion-induced animal model, immunohistochemical analyses of SMemb, neutrophil elastase, angiotensinogen, AT1 receptor, MDA, and ET-1 were performed to elucidate the precise effect of hypoperfusion on the aortic wall on 28 days after the induction of hypoperfusion (Figure 1 and Figure S2). Positive areas for SMemb+ cells (synthetic SMCs), neutrophil elastase, angiotensinogen, AT1 receptor, MDA, and ET-1 were significantly increased in the hypoperfusion-induced AAA wall (Figure 1).

### 3.2. Time-Dependent Changes in AAA-Related Factors in the Hypoperfusion-Induced Animal Model

**Figure 2:**
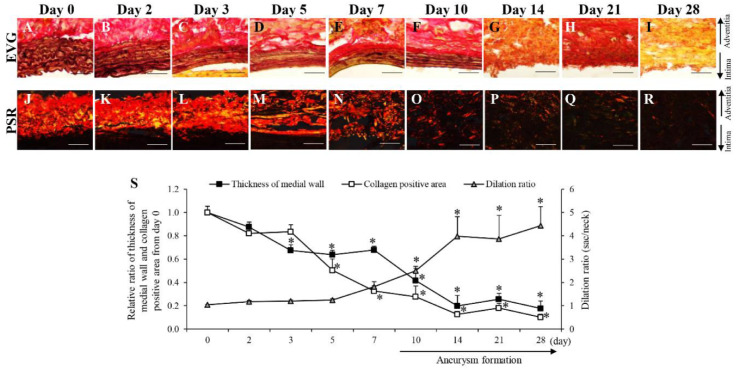
Time-dependent changes of thickness of medial wall and collagen positive area. *(***A**–**I**) Representative images of the Elastica van Gieson (EVG) staining (scale bar = 50 µm). (**J**–**R**) Representative images of the picrosirius red (PSR) staining (scale bar = 50 µm). (**S**) Quantification of the relative ratio from day 0 of thickness of medial wall, collagen positive area and dilation ratio in the vascular wall. Data are expressed as the mean ± SEM. * *P* < 0.05 versus day 0. Day 0 (n = 7), day 2 (n = 6), day 3 (n = 5), day 5 (n = 6), day 7 (n = 6), day 10 (n = 5), day 14 (n = 6), day 21 (n = 14), and day 28 (n = 7).

Next, we performed a time-dependent pathological analysis of AAA-related factors in the aortic wall in the hypoperfusion-induced animal model. The thickness of the medial wall significantly decreased from the 3rd to the 28th day after aortic wall ligation to induce hypoperfusion (Figure 2A–I,S). The collagen-positive area significantly decreased from the 5th to the 28th day after aortic wall ligation (Figure 2J–S). AAA formation was observed from the 10th to the 28th day after the aortic wall ligation (Figure 2S and Figure S3).

**Figure 3:**
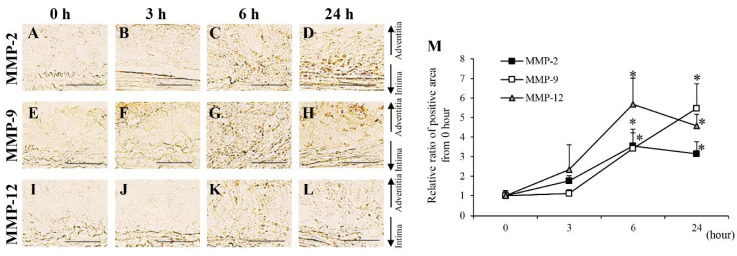
Time-dependent changes of matrix metalloproteinase (MMP)-2, MMP-9 and MMP-12 from 0 to 24 h. (**A**–**D**) Representative images of the immunostaining for MMP-2 (scale bar = 50 µm). (**E**–**H**) Representative images of the immunostaining for MMP-9 (scale bar = 50 µm). (**J**–**L**) Representative images of the immunostaining for MMP-12 (scale bar = 50 µm). (**M**) Quantification of the relative ratio from 0 h of the areas positive for MMP-2, MMP-9, and MMP-12 in the vascular wall. Data are expressed as the mean ± SEM. *P < 0.05 versus 0 h. MMP-2: 0 h (n = 6), 3 h (n = 7), 6 h (n = 5), and 24 h (n = 7), MMP-9: 0 h (n = 6), 3 h (n = 5), 6 h (n = 6), and 24 h (n = 6), and MMP-12: 0 h (n = 6), 3 h (n = 7), 6 h (n = 6), and 24 h (n = 7).

**Figure 4:**
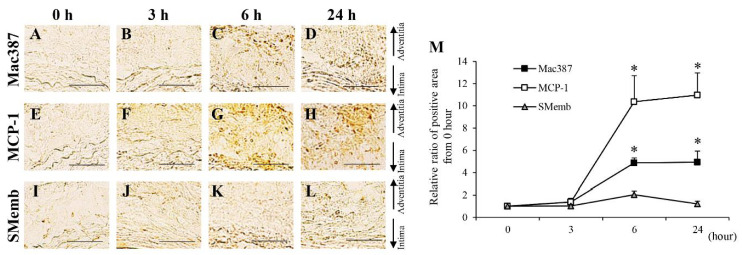
Time-dependent changes of mac387^+^ macrophage, monocyte chemoattractant protein-1 (MCP-1) and synthetic vascular smooth muscle (SMemb) from 0 to 24 h. (**A**–**D**) Representative images of the immunostaining for mac387 (scale bar = 50 µm). (**E**–**H**) Representative images of the immunostaining for MCP-1 (scale bar = 50 µm). (**J**–**L**) Representative images of the immunostaining for SMemb (scale bar = 50 µm). (**M**) Quantification of the relative ratio from 0 h of the areas positive for mac387, MCP-1, and SMemb in the vascular wall. Data are expressed as the mean ± SEM. * *P* < 0.05 versus 0 h. Mac387: 0 h (n = 5), 3 h (n = 6), 6 h (n = 5), and 24 h (n = 5), MCP-1: 0 h (n = 6), 3 h (n = 5), 6 h (n = 6), and 24 h (n = 6), and SMemb: 0 h (n = 6), 3 h (n = 6), 6 h (n = 6), and 24 h (n = 6).

**Figure 5:**
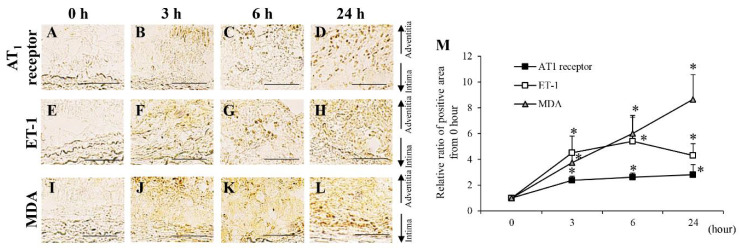
Time-dependent changes of angiotensin II type 1 (AT_1_) receptor, endothelin-1 (ET-1) and malondialdehyde (MDA) from 0 to 24 h. (**A**–**D**) Representative images of the immunostaining for AT_1_ receptor (scale bar = 50 µm). (**E**–**H**) Representative images of the immunostaining for ET-1 (scale bar = 50 µm). (**J**–**L**) Representative images of the immunostaining for MDA (scale bar = 50 µm). (**M**) Quantification of the relative ratio from 0 h of the areas positive for AT_1_ receptor, ET-1, and MDA in the vascular wall. Data are expressed as the mean ± SEM. * *P* < 0.05 versus 0 h. AT_1_ receptor: 0 h (n = 6), 3 h (n = 6), 6 h (n = 5), and 24 h (n = 7), ET-1: 0 h (n = 6), 3 h (n = 7), 6 h (n = 5), and 24 h (n = 7), and MDA: 0 h (n = 6), 3 h (n = 6), 6 h (n = 6), and 24 h (n = 7).

The areas positive for MMP-2, MMP-9, and MMP-12 were significantly increased from the 2nd to the 28th day after aortic wall ligation (Figures S4 and S5A–I,S). To investigate their expression within a short period, time-dependent analyses of MMP-2, MMP-9, and MMP-12 from 0 to 24 h after aortic wall ligation were performed (Figure 3). Areas positive for MMP-2, MMP-9, and MMP-12 significantly increased from 6 h after aortic wall ligation (Figure 3). The neutrophil elastase-positive area significantly increased from the 3rd to the 28th day after aortic wall ligation (Figure S4J–S). Mac387+ cells (M1 macrophages) showed a significant increase from the 2nd to the 28th day after aortic wall ligation (Figure S6A–I,S). Moreover, Mac387+ cells significantly increased from 6 h after aortic wall ligation (Figure 4A–D,M). CD163+ cells (M2 macrophages) significantly increased from the 21st to the 28th day after aortic wall ligation (Figure S6J–S). The area positive for MCP-1 significantly increased from the 2nd to the 28th day after the induction of hypoperfusion and significantly increased from 6 h after aortic wall ligation (Figure S7 and Figure 4E–H,M). Alpha-SMA+ cells (contractile SMCs) in the medial wall significantly decreased from the 3rd to the 28th day after aortic wall ligation (Figure S8A–I,S). Conversely, SMemb+ cells (synthetic SMCs) significantly increased from the 2nd to the 28th day after aortic wall ligation (Figure S8J–S). The positive area for SMemb+ cells did not change until 24 h (Figure 4I–M). The positive areas for angiotensinogen significantly increased from the 3rd to the 28th day after aortic wall ligation (Figure S9A–I, S). The positive areas for AT1 receptor, ET-1, and MDA were significantly increased from the 2nd to the 28th day after aortic wall ligation (Figures S9J–S and S10). In addition, the positive areas for the AT1 receptor, ET-1, and MDA significantly increased at 3 h after aortic wall ligation (Figure 5).

### 3.3. Colocalization Studies of AAA-Related Factors with α-SMA+ Cells and Mac387+ Cells

**Figure 6:**
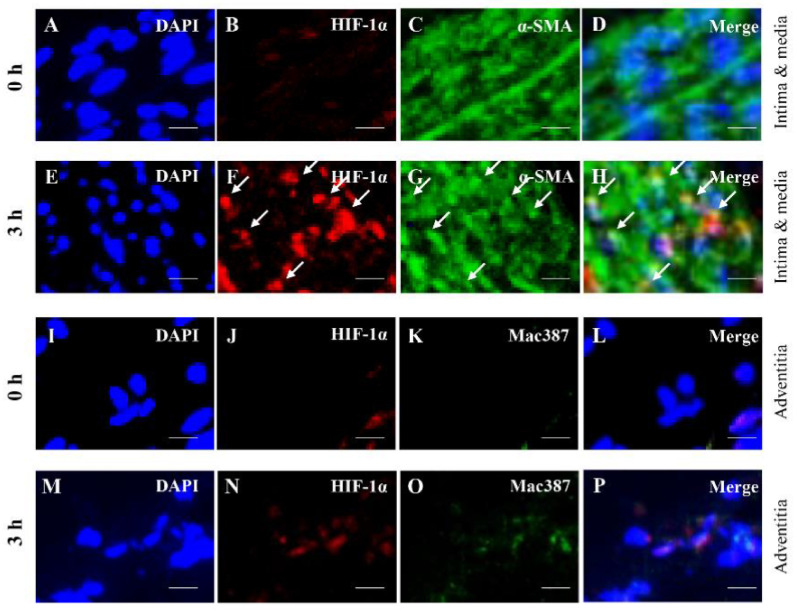
Localization of hypoxia inducible factor (HIF)-1α, α-smooth muscle actin (α-SMA) ^+^ cells and mac387^+^ macrophages in the aortic wall. Double-immunostaining for HIF-1α and α-SMA in 0 h (**A**–**D**) and 3 h (**E**–**H**). The white arrows indicate the co-localized region. Double-immunostaining for HIF-1α and mac387^+^ macrophages in 0 h (**I**–**L**) and 6 h (**M**–**P**). Scale bar = 15 µm. 0 h (n = 5), 3 h (n = 5), and 6 h (n = 5).

To understand the AAA pathology at the initial stage, we investigated the colocalization of AAA-related factors with α-SMA+ cells and Mac387+ cells. Mac387+ cells were significantly detected at 6 h after induction of hypoperfusion and were analyzed in 6 h. HIF-1α was colocalized with α-SMA+ cells in 3 h, but not in Mac387+ cells (Figure 6). AT1 receptor was colocalized with α-SMA+ cells both at 0 h and 3 h, and with Mac387+ cells in 6 h (Figure S11). ET-1 and MDA were colocalized with α-SMA+ cells in 3 h, but not in Mac387+ cells (Figures S12 and S13). MMP-2 was colocalized with α-SMA+ cells both at 0 and 3 h, but not with Mac387+ cells (Figure S14). MMP-9 was colocalized with α-SMA+ cells in both 3 h and Mac387+ cells at 6 h (Figure S15). MMP-12 did not colocalize with α-SMA+ cells at both 0 h and 3 h, but did with Mac387+ cells in 6 h (Figure S16). MCP-1 was colocalized with α-SMA+ cells both at 0 and 3 h, but not with Mac387+ cells (Figure S17). Neutrophil elastase did not colocalize with either α-SMA+ or Mac387+ cells (Figure S18). Angiotensinogen was colocalized with α-SMA+ cells both at 0 h and 3 h, but not with Mac387+ cells (Figure S19). The data are summarized in Table 1.

## 4. Discussion

In this study, we performed time-dependent pathological analyses of the hypoperfusion-induced aortic walls to clarify the molecular mechanisms correlating hypoperfusion and AAA formation. A comparison of the pathological events of a hypoperfusion-induced AAA animal model and human AAA is shown in Table 2. Time-dependent changes in pathologies and proteins, including those in previous studies, were sorted by the dynamically changing time of each factor (Table 3A,B). Five dynamic changes were observed in the evaluated AAA-related factors after the induction of hypoperfusion (Table 3B).

Levels of the AT1 receptor, ET-1, and MDA initially increased 3 h after the induction of hypoperfusion. The AT1 receptor is required for AngII-induced AAA formation [45]. AT1 receptors, including endothelial cells, SMCs, and macrophages, are detected in several cells of the arterial wall. AngII stimulates MCP-1 secretion [46], ROS production [47], and MMP-2 expression in SMCs [48]. ET-1 is a 21-amino-acid peptide and is one of the most potent endogenous vasoconstrictors. ET-1 overexpression induces aneurysms in apolipoprotein E knockout mice with increased oxidative stress levels and monocyte/macrophage infiltration [49]. Similarly, plasma ET-1 levels [50,51] and aortic tissue AT1 receptor levels [43] are significantly increased in patients with AAA. MDA is an oxidative stress marker in AAA walls. Oxidative stress due to ROS production is involved in vascular injury and AAA development [52]. Oxidative stress is induced by endothelial dysfunction and nicotinamide adenine dinucleotide phosphate oxidase overexpression [53]. In our previous report, HIF-1α levels were also increased 3 h after the induction of hypoperfusion [30]. HIF-1α is reportedly the upstream factor of the molecules involved in AAA development [22], including ET-1 [54]. These data suggest that hypoperfusion in the aortic wall first induces hypoxia and vasoconstriction-related factors (AT1 receptor and ET-1) related to increased oxidative stress and inflammation.

Second, 6 h after the induction of hypoperfusion, the levels of MMP-2, MMP-9, MMP-12, Mac387+ macrophages (M1 macrophages), and MCP-1 significantly increased in the aortic walls. Increased levels of these AAA-related factors are consistent with findings in human AAA [17,23,24,26,37,40]. Oxidative stress due to ROS production in vascular SMCs reportedly induces excess MMP-2 expression [55,56]. In addition, HIF-1α induces MMP expression under hypoxic conditions [57]. As mentioned above, the increased levels in the second group can be associated with increased levels of factors in the first group and HIF-1α.

Third, synthetic SMC, SMemb+ SMC, significantly increased 2 days after the induction of hypoperfusion. SMemb is a non-muscle myosin heavy chain abundantly expressed in SMCs in the immature aorta [58], and an increased level of SMemb+ SMC is consistent with human AAA [41]. The increased level of synthetic SMC may be associated with the decreased level of contractile SMC in the fourth group. A hypoperfusion-induced AAA model may reproduce both the increase in synthetic SMC and the decreased in contractile SMC observed in human AAA. Contractile SMCs regulate the diameter and blood flow of the normal aorta. In response to aortic injury, contractile SMC shifts the aorta from a contractile state to a synthetic state, known as “phenotypic switching” [59]. Synthetic SMCs can secrete various extracellular matrix proteins and MMP molecules. Phenotypic switching is involved in the development of AAA [41,60]. Ailawadi et al. reported that phenotypic switching is an early event in aortic aneurysms formed in an elastase-induced AAA animal model [61]. The same group reported the possibility that Krüppel-like factor 4 (KLF4) regulates SMC phenotype switching [60]. KLF4 is able to induce pluripotent stem cells [62] and HIF-1α reportedly induces its expression in cancer cells [63], suggesting that hypoperfusion might be involved in KLF4 expression in the AAA wall.

Fourth, neutrophil elastase, angiotensinogen, and contractile SMC significantly changed 3 days after the induction of hypoperfusion. Changes in these AAA-related factors are consistent with those observed in human AAA [27,42,43]. Neutrophil elastase, which is released by activated neutrophils, plays an important role in the development of AAA [64,65]. It reportedly regulates the formation of neutrophil extracellular traps (NETs) [66], which play a critical role in the neutrophil-mediated development of AAA [67]. In sepsis-induced thrombus formation, HIF-1α activation is associated with NET formation during thrombosis [68], suggesting that NETs are downstream factors of hypoperfusion. Angiotensinogen is involved in hypertension as a precursor of AngII [69]. The promoter domain of angiotensinogen contains a binding site for HIF-1α, which mediates the transcriptional activation of angiotensinogen [70]. A decreased level of contractile SMC might be associated with the phenotypic switching mentioned above. 

**Figure 7:**
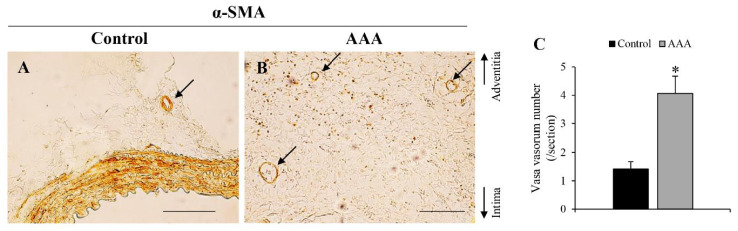
Observation of adventitial vasa vasorum (VV). Representative images of immunostaining for α-smooth muscle actin in (**A**) control wall (scale bar = 100 μm). (**B**) AAA wall (scale bar = 200 μm). (**C**) VV number (/section) in the aortic wall. *P < 0.05 versus control wall. Control wall (n = 12) and AAA wall (n = 17).

Fifth, the number of M2 macrophages (CD163+ cells) significantly increased 21 days after AAA formation. The observation of M2 macrophages is consistent with the findings of a previous human AAA study [25]. M2 macrophages promote anti-inflammatory response [71] and angiogenesis [72]. Adventitial angiogenesis has been observed in human AAA [22,32]. In this experimental model, the VV count in the adventitial wall increased in the AAA wall (Figure 7 and Table 2A). The increased VV count might indicate a compensatory reaction to attenuate the hypoxic conditions in the AAA wall. HIF-1α stimulates angiogenesis via the NF-E2–related factor 2/heme oxygenase-1 (HO-1) pathway [72]. HO-1 is involved in promoting macrophage differentiation into the M2 phenotype as well as other inducible factors, such as interleukins and micro RNAs [73,74].

Colocalization studies have suggested the mechanisms underlying the cascades of AAA pathology in the initial stage (0 to 6 h). HIF-1α was colocalized with α-SMA+ cells in 3 h, but not with Mac387+ cells, suggesting that hypoperfusion-induced hypoxia directly affects the activities of contractile SMCs in the initial stage of AAA. The expression of ET-1, MDA, and MMP-9 in contractile SMCs might be associated with hypoperfusion-induced expression of HIF-1α. 

In this study, we showed the characteristic dynamics of human AAA-related factors using a hypoperfusion-induced animal model. To our knowledge, this is the first time-dependent pathological study of an AAA animal model. Expression of HIF-1α and several AAA-related factors was observed in contractile SMCs in the initial stage after induction of hypoperfusion. In addition, the pathological features of hypoperfusion-induced AAA in this study were consistent with those of human AAA. This study suggested that hypoperfusion could induce AAA-related factors, as reported in human AAA studies. However, the limitations of this study should be noted. Because this study was observational, the interrelation between these factors was not determined. The time lag of the dynamic pattern of HIF-1α-related factors suggests the existence of an unidentified interactional cascade. Further studies are required to elucidate these points.

## 5. Conclusions

Time-dependent pathological analysis is important to understand the mechanism underlying AAA formation and development. We found that five groups were formed by dynamically changing the time. VV stenosis, as an inducer of increased HIF-1α levels, may be a new drug development target. Our time-dependent pathological analysis clarified the black box of AAA pathology.

## Figures and Tables

**Table 1:** Localization of abdominal aortic aneurysm-related factors.

	Contractile SMC (α-SMA)		M1 Macrophage (Mac387)	
	0 h	3 h	0 h	6 h
HIF-1α	-	+	-	-
AT_1_ receptor	+	+	-	+
ET-1	-	+	-	-
MDA	-	+	-	-
MMP-2	+	+	-	-
MMP-9	-	+	-	+
MMP-12	-	-	-	+
MCP-1	+	+	-	-
Neutrophil elastase	-	-	-	-
Angiotensinogen	+	+	-	-

+: co-localization in the aortic wall.

**Table 2:** Comparison of pathological features between hypoperfusion-induced model and human abdominal aortic aneurysm (AAA).

(**A**) Pathological events in human AAA and hypoperfusion-induced model.								
**Pathological feature**		**Human AAA**		**Reference**		**Hypoperfusion model**		**Reference**
Aortic dilation		+		*Lancet* [10]		+		*PLoS ONE* [8]
Aneurysm rupture		+		*Lancet* [10]		+		*Sci Rep* [9], *J Oleo Sci* [21]
Adipogenesis		+		*J Vasc Res* [13], *J Vasc Surg.* [11], *Acta Biomater* [12]		+		*Sci Rep* [9], *J Oleo Sci* [21], *Adipocyte* [30]
Intraluminal thrombus		+		*J Vasc Surg.* [14], *J Vasc Surg.* [6]		+		*PLoS ONE* [8]
Vascular wall thickening		+		*J Surg. Case Rep* [15]		+		*Sci Rep* [9]
Medial wall thinning		+		*Circulation* [16]		+		*Sci Rep* [9]
Collagen fiber degradation		+		*Lancet* [10]		+		*PLoS ONE* [8]
Elastin fiber degradation		+		*Lancet* [10]		+		*PLoS ONE* [8]
Gelatinolytic activity		+		*J Vasc Surg.* [17], *World J Surg.* [18]		+		*PLoS ONE* [8]
Oxidative stress		+		*Clin Sci (Long)* [19], *In J Cardiol* [20]		+		*Biosci Biotechnol Biochem* [31]
Stenosis of vasa vasorum		+		*PLoS ONE* [7]		+		*PLoS ONE* [8]
Angiogenesis		+		*J Am Heart Assoc* [22], *PLoS ONE* [32]		+		Figure 7
Atherosclerosis		+		*Curr Opin Cardiol* [33]		-		
Calcification		+		*Int Angiol* [34], *Atherosclerosis* [35], *Circ J* [36]		-		
(**B**) Protein expressions of AAA-related factors in human AAA and hypoperfusion-induced model.								
**Pathological feature**	**Human AAA**		**Reference**		**Hypoperfusion model**		**Reference**	
HIF-1α	↑		*J Am Heart Assoc* [22]		↑		*PLoS ONE* [8]	
MMP-2	↑		*J Vasc Surg.* [17], *Ann N Y Acad Sci* [37]		↑		*PLoS ONE* [8], *Sci Rep* [9], *J Vasc Res* [38], *J Oleo Sci* [39]	
MMP-9	↑		*J Vasc Surg.* [17], *Ann N Y Acad Sci* [37]		↑		*PLoS ONE* [8], *Sci Rep* [9], *J Vasc Res* [38], *J Oleo Sci* [39]	
MMP-12	↑		*J Clin Invest* [23]		↑		*J Vasc Res* [38], *J Oleo Sci* [39]	
M1 macrophage	↑		*Dis Markers* [40], *Arterioscler Thromb Vasc Biol* [24]		↑		*PLoS ONE* [8], *Sci Rep* [9], *J Vasc Res* [38], *J Oleo Sci* [39]	
M2 macrophage	↑		*Int J Cardiol* [25]		↑		*Sci Rep* [9]	
MCP-1	↑		*Am J Pathol* [26]		↑		*Sci Rep* [9], *J Vasc Res* [38], *J Oleo Sci* [39]	
Contractile SMC	↓		*Arterioscler Thromb Vasc Biol* [27]		↓		*PLoS ONE* [8], *Sci Rep* [9]	
Synthetic SMC	↑		*Cardiovasc Pathol* [41]		↑		Figure 1C	
Neutrophil elastase	↑		*Cardiovasc Surg.* [42]		↑		Figure 1F	
Angiotensinogen	↑		*Atherosclerosis* [43]		↑		Figure 1I	
AT_1_ receptor	↑		*Atherosclerosis* [43]		↑		Figure 1L	
MDA	↑		*Arterioscler Thromb Vasc Biol* [44]		↑		Figure 1O	
ET-1	?				↑		Figure 1R	

‘→’ indicates no difference in sac wall compared to neck wall. ‘↑’ or ‘↓’ indicates *P* < 0.05 versus neck wall. ‘↑’ indicates significant increase and ‘↓’ indicates significant decrease in sac wall compared to neck wall (*P* < 0.05 versus neck wall). HIF-1α: hypoxia-inducible factor-1α, MMP: matrix metalloproteinase, MCP-1: monocyte chemoattractant protein-1, SMC: smooth muscle cell, α-SMA: α-smooth muscle actin, AT_1_: angiotensin II Type 1, MDA: malondialdehyde, ET-1: endothelin-1.

**Table 3:** Time-dependent changes of abdominal aortic aneurysm-related factors in hypoperfusion-induced model.

(**A**) Time-dependent changes of pathological events in hypoperfusion-induced model.													
**Pathology**	**Day 1**												
	**0 h**	**3 h**	**6 h**	**24 h**	**Day 2**	**Day 3**	**Day 5**	**Day 7**	**Day 10**	**Day 14**	**Day 21**	**Day 28**	
Medial wall thickness	→	→	→	→	→	↓	↓	↓	↓	↓	↓	↓	
Collagen fiber degradation	→	→	→	→	→	→	↑	↑	↑	↑	↑	↑	
AAA	→	→	→	→	→	→	→	→	↑	↑	↑	↑	
(**B**) Time-dependent changes of protein expressions of AAA-related factors in hypoperfusion-induced model.													
**Protein**	**Day 1**												**Group**
	**0 h**	**3 h**	**6 h**	**24 h**	**Day 2**	**Day 3**	**Day 5**	**Day 7**	**Day 10**	**Day 14**	**Day 21**	**Day 28**	
HIF-1α	→	↑	↑	↑	↑	↑	↑	↑	↑	↑	↑	↑	First
AT_1_ receptor	→	↑	↑	↑	↑	↑	↑	↑	↑	↑	↑	↑	
ET-1	→	↑	↑	↑	↑	↑	↑	↑	↑	↑	↑	↑	
MDA	→	↑	↑	↑	↑	↑	↑	↑	↑	↑	↑	↑	
MMP-2	→	→	↑	↑	↑	↑	↑	↑	↑	↑	↑	↑	Second
MMP-9	→	→	↑	↑	↑	↑	↑	↑	↑	↑	↑	↑	
MMP-12	→	→	↑	↑	↑	↑	↑	↑	↑	↑	↑	↑	
M1 macrophage (Mac387)	→	→	↑	↑	↑	↑	↑	↑	↑	↑	↑	↑	
MCP-1	→	→	↑	↑	↑	↑	↑	↑	↑	↑	↑	↑	
Synthetic SMC (SMemb)	→	→	→	→	↑	↑	↑	↑	↑	↑	↑	↑	Third
Neutrophil elastase	→	→	→	→	→	↑	↑	↑	↑	↑	↑	↑	Forth
Angiotensinogen	→	→	→	→	→	↑	↑	↑	↑	↑	↑	↑	
Contractile SMC (α-SMA)	→	→	→	→	→	↓	↓	↓	↓	↓	↓	↓	
M2 macrophage (CD163)	→	→	→	→	→	→	→	→	→	→	↑	↑	Fifth

‘↑’ or ‘↓’ indicates *P* < 0.05 versus day 0 (pre-induction of hypoperfusion). HIF-1α: hypoxia-inducible factor-1α, MMP: matrix metalloproteinase, MCP-1: monocyte chemoattractant protein-1, SMC: smooth muscle cell, α-SMA: α-smooth muscle actin, AT_1_: angiotensin II Type 1, ET-1: endothelin-1, MDA: malondialdehyde.

## Data Availability

Data of this study are available from the corresponding author.

## Supplementary Materials

The following are available online at https://www.mdpi.com/2079-7737/10/2/149/s1, Figure S1: Induction of abdominal aortic wall hypoperfusion; Figure S2: Immunohistochemical staining for synthetic vascular smooth muscle (SMemb), neutrophil elastase, angiotensinogen, angiotensin II type 1 (AT1) receptor, malondialdehyde (MDA) and endothelin-1 (ET-1); Figure S3: Time-dependent changes of the abdominal aortic aneurysm dilation; Figure S4: Time-dependent changes of matrix metalloproteinase (MMP) -2 and MMP-9 from day 0 to 28; Figure S5: Time-dependent changes of matrix metalloproteinase (MMP) -12 and neutrophil elastase from day 0 to 28; Figure S6: Time-dependent changes of mac387+ and CD163+ macrophages from day 0 to 28; Figure S7: Time-dependent change of monocyte chemoattractant protein-1 (MCP-1) from day 0 to 28; Figure S8: Time-dependent changes of contractile vascular smooth muscle (α-smooth muscle actin (α-SMA)) and synthetic vascular smooth muscle (SMemb) from day 0 to 28; Figure S9: Time-dependent changes of angiotensinogen, and angiotensin II type 1 (AT1) receptor from day 0 to 28; Figure S10: Time-dependent changes of endothelin-1 (ET-1) and malondialdehyde (MDA) from day 0 to 28; Figure S11: Localization of angiotensin II type 1 (AT1) receptor, α-smooth muscle actin (α-SMA) + cells and mac387+ macrophages in the aortic wall; Figure S12: Localization of endothelin-1 (ET-1), α-smooth muscle actin (α-SMA) + cells and mac387+ macrophages in the aortic wall; Figure S13: Localization of malondialdehyde (MDA), α-smooth muscle actin (α-SMA) + cells and mac387+ macrophages in the aortic wall; Figure S14: Localization of matrix metalloproteinase (MMP) -2, α-smooth muscle actin (α-SMA) + cells and mac387+ macrophages in the aortic wall; Figure S15: Localization of matrix metalloproteinase (MMP) -9, α-smooth muscle actin (α-SMA) + cells and mac387+ macrophages in the aortic wall; Figure S16: Localization of matrix metalloproteinase (MMP) -12, α-smooth muscle actin (α-SMA) + cells and mac387+ macrophages in the aortic wall; Figure S17: Localization of monocyte chemoattractant protein-1 (MCP-1), α-smooth muscle actin (α-SMA) + cells and mac387+ macrophages in the aortic wall; Figure S18: Localization of neutrophil elastase, α-smooth muscle actin (α-SMA) + cells and mac387+ macrophages in the aortic wall; Figure S19: Localization of angiotensinogen, α-smooth muscle actin (α-SMA) + cells and mac387+ macrophages in the aortic wall.
