# Supplementary material for: Time-Dependent Pathological Changes in Hypoperfusion-Induced Abdominal Aortic Aneurysm
Source: Biology (Basel). 2021 Feb 14;10(2):149. doi: 10.3390/biology10020149 (PMC7917844; doi:10.3390/biology10020149)
Supplement: Supplementary file 1 [file biology-10-00149-s001.zip › 210127 Supplemental Materials.docx]

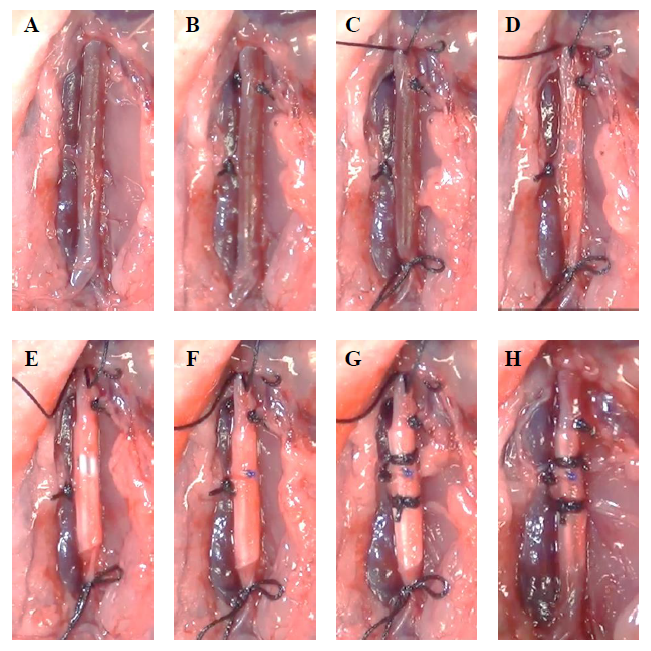


**Figure S1:** Induction of abdominal aortic wall hypoperfusion. (**A**) The infrarenal aorta is exfoliated from the perivascular tissue. (**B**) Vessels branching from the abdominal aorta are ligated, and (**C**) the abdominal aorta is ligated to block the aortic blood flow. (**D**) Small incision was created, and (**E**) plastic catheter shortened to 9 mm long is inserted via a small incision. (**F**) The incision is repaired, and (**G**) the abdominal aorta is ligated together with the plastic catheter. (**H**) The string blocking the blood flow in the aorta is untied.


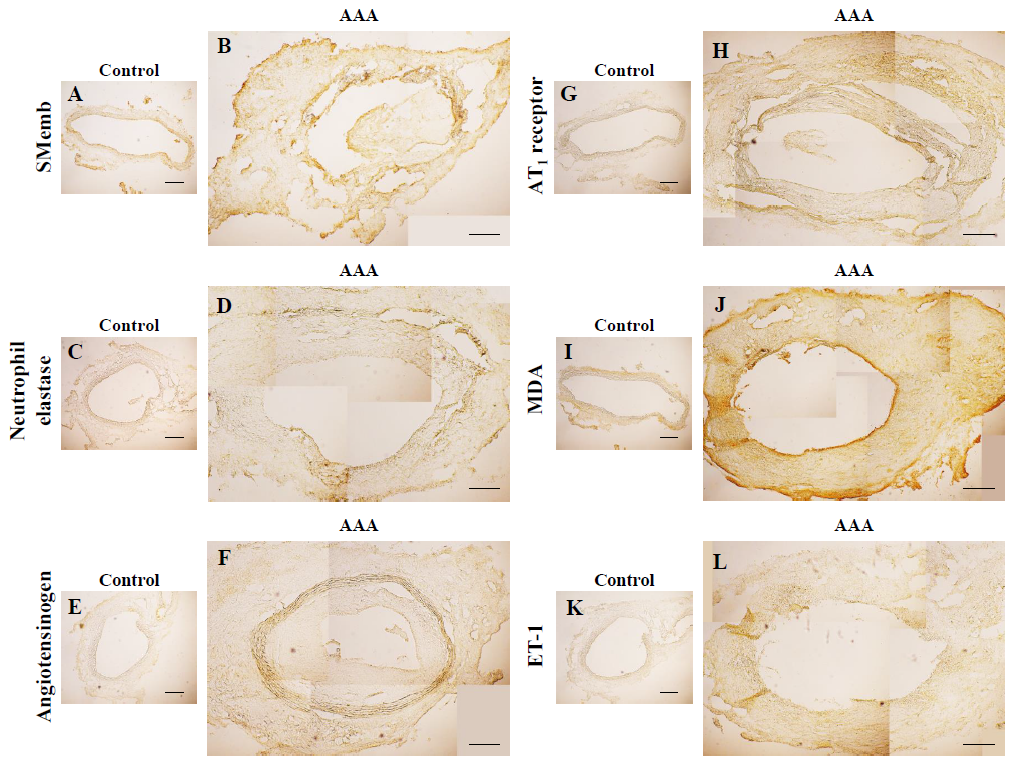


**Figure S2:** Immunohistochemical staining for synthetic vascular smooth muscle (SMemb), neutrophil elastase, angiotensinogen, angiotensin II type 1 (AT_1_) receptor, malondialdehyde (MDA) and endothelin-1 (ET-1). (**A**, **B**) Representative images of the immunostaining for SMemb. (**C**, **D**) Representative images of the immunostaining for neutrophil elastase. (**E**, **F**) Representative images of the immunostaining for angiotensinogen. (**G**, **H**) Representative images of the immunostaining for AT_1_ receptor. (**I**, **J**) Representative images of the immunostaining for MDA. (**K**, L) Representative images of the immunostaining for ET-1. scale bar = 200 µm.


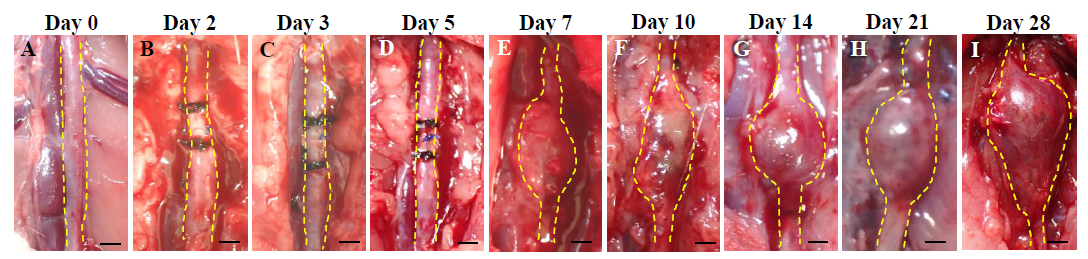


**Figure S3:** Time-dependent changes of the abdominal aortic aneurysm dilation. (**A–I**) Representative images of the abdominal aorta on days 0, 2, 3, 5, 7, 10, 14, 21, and 28 after the induction of hypoperfusion (scale bar = 1.5 mm). Day 0 (n = 7), day 2 (n = 6), day 3 (n = 5), day 5 (n = 6), day 7 (n = 6), day 10 (n = 5), day 14 (n = 6), day 21 (n = 14) and day 28 (n = 7).


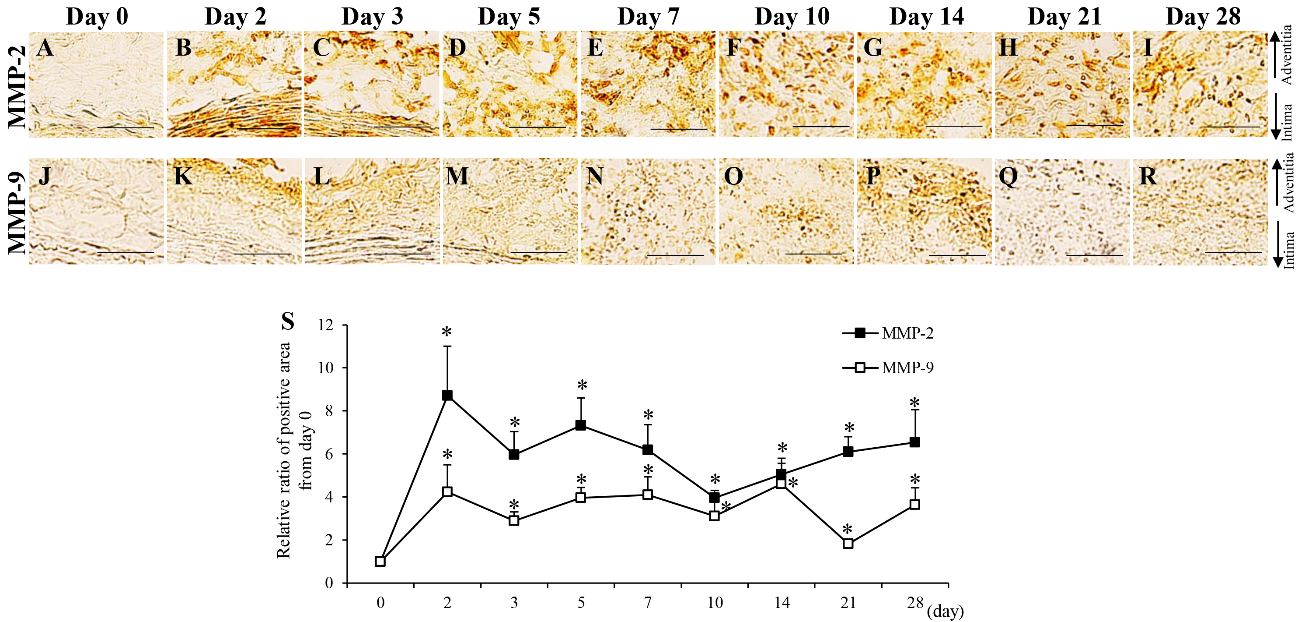


**Figure S4:** Time-dependent changes of matrix metalloproteinase (MMP) -2 and MMP-9 from day 0 to 28. (**A**-**I**) Representative images of the immunostaining for MMP-2 (scale bar = 50 µm). (**J**-**R**) Representative images of the immunostaining for MMP-9 (scale bar = 50 µm). (**S**) Quantification of the relative ratio from day 0 of areas positive for MMP-2 and MMP-9 in the vascular wall. Data are expressed as the mean ± SEM. **P* < 0.05 versus day 0. MMP-2: day 0 (n = 6), day 2 (n = 5), day 3 (n = 8), day 5 (n = 6), day 7 (n = 5), day 10 (n = 6), day 14 (n = 6), day 21 (n = 11) and day 28 (n = 6), and MMP-9: day 0 (n = 6), day 2 (n = 5), day 3 (n = 6), day 5 (n = 7), day 7 (n = 5), day 10 (n = 6), day 14 (n = 6), day 21 (n = 12) and day 28 (n = 6).


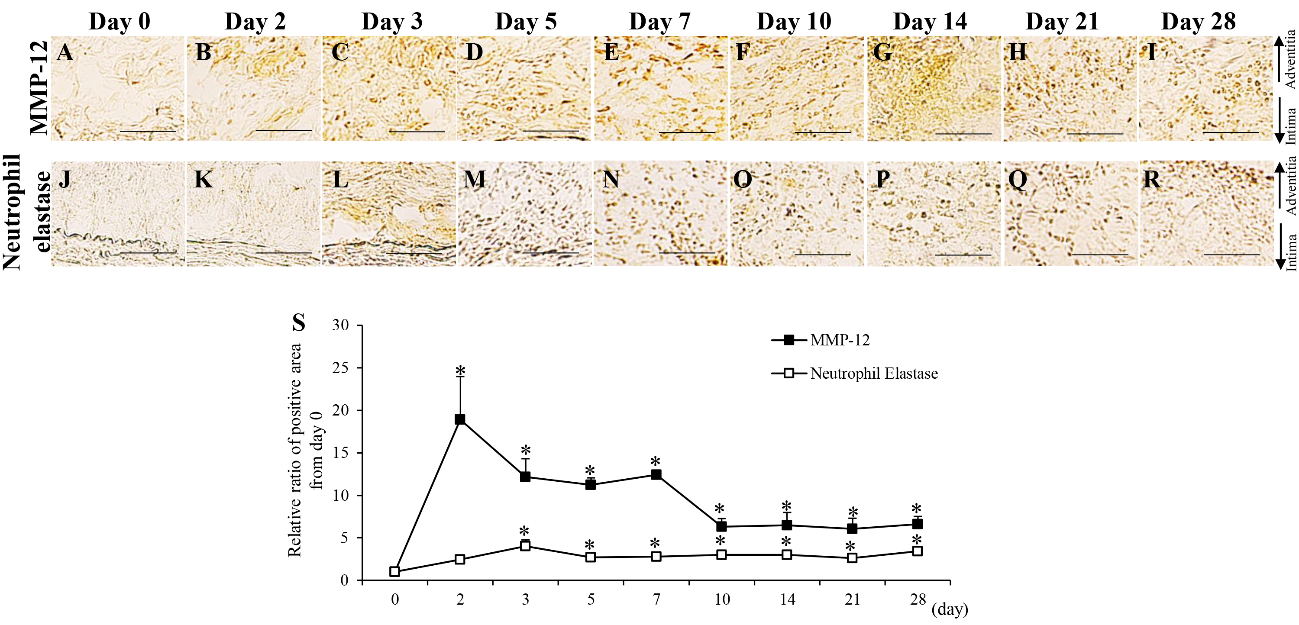


**Figure S5:** Time-dependent changes of matrix metalloproteinase (MMP) -12 and neutrophil elastase from day 0 to 28. (**A**-**I**) Representative images of the immunostaining for MMP-12 (scale bar = 50 µm). (**J**-**R**) Representative images of the immunostaining for neutrophil elastase (scale bar = 50 µm). (**S**) Quantification of the relative ratio from day 0 of areas positive for MMP-12 and neutrophil elastase in the vascular wall. Data are expressed as the mean ± SEM. **P* < 0.05 versus day 0. MMP-12: day 0 (n = 5), day 2 (n = 6), day 3 (n = 6), day 5 (n = 5), day 7 (n = 5), day 10 (n = 6), day 14 (n = 6), day 21 (n = 12) and day 28 (n = 6), and neutrophil elastase: day 0 (n = 6), day 2 (n = 6), day 3 (n = 8), day 5 (n = 7), day 7 (n = 7), day 10 (n = 6), day 14 (n = 6), day 21 (n = 12) and day 28 (n = 6).


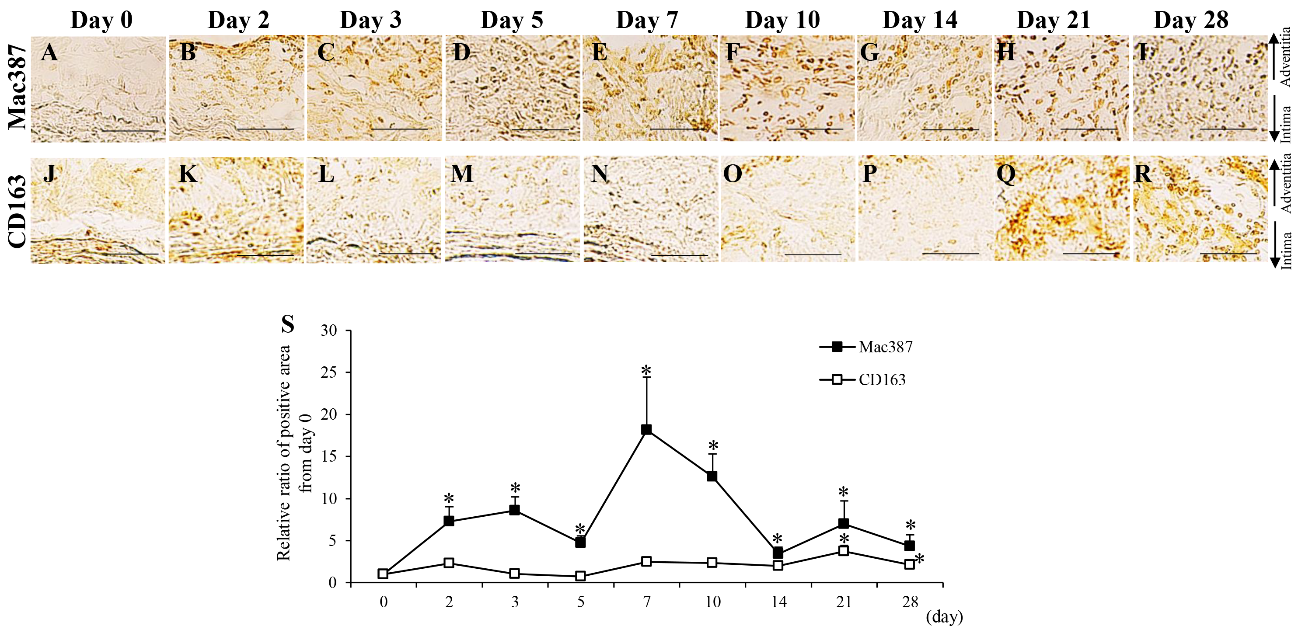


**Figure S6:** Time-dependent changes of mac387^+^ and CD163^+^ macrophages from day 0 to 28. (**A**-**I**) Representative images of the immunostaining for mac387 (scale bar = 50 µm). (**J**-**R**) Representative images of the immunostaining for CD163 (scale bar = 50 µm). (**S**) Quantification of the relative ratio from day 0 of areas positive for mac387 and CD163 in the vascular wall. Data are expressed as the mean ± SEM. **P* < 0.05 versus day 0. Mac387: day 0 (n = 6), day 2 (n = 6), day 3 (n = 5), day 5 (n = 7), day 7 (n = 7), day 10 (n = 6), day 14 (n = 5), day 21 (n = 5) and day 28 (n = 8), and CD163: day 0 (n = 6), day 2 (n = 6), day 3 (n = 5), day 5 (n = 7), day 7 (n = 7), day 10 (n = 6), day 14 (n = 5), day 21 (n = 5) and day 28 (n = 7).


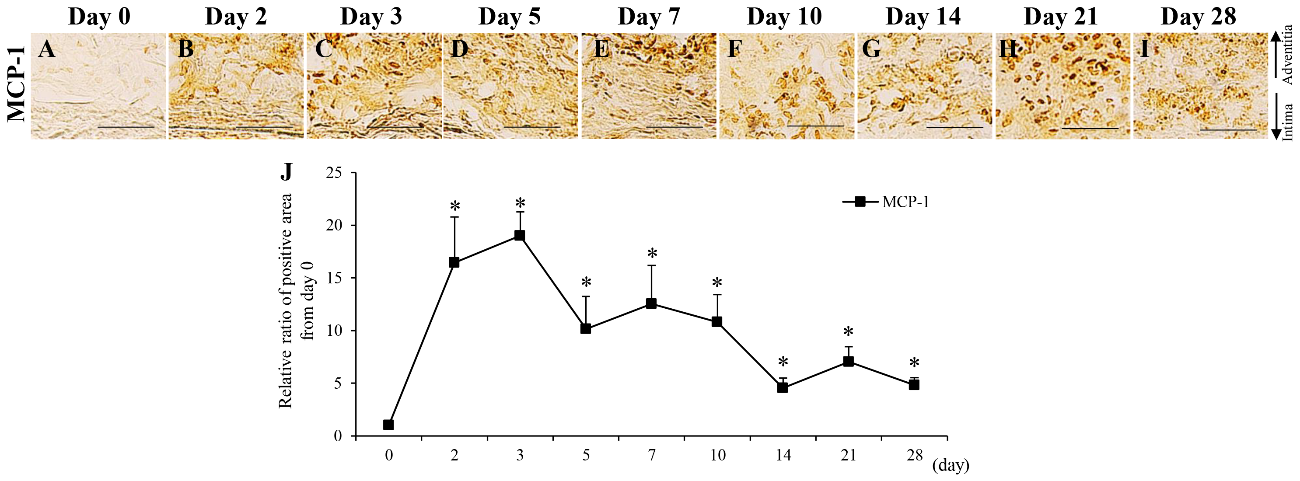


**Figure S7:** Time-dependent change of monocyte chemoattractant protein-1 (MCP-1) from day 0 to 28. (**A**-**I**) Representative images of the immunostaining for MCP-1 (scale bar = 50 µm). (**J**) Quantification of the relative ratio from day 0 of areas positive for MCP-1 in the vascular wall. Data are expressed as the mean ± SEM. **P* < 0.05 versus day 0. MCP-1: day 0 (n = 6), day 2 (n = 6), day 3 (n = 5), day 5 (n = 7), day 7 (n = 7), day 10 (n = 6), day 14 (n = 5), day 21 (n = 5) and day 28 (n = 8).


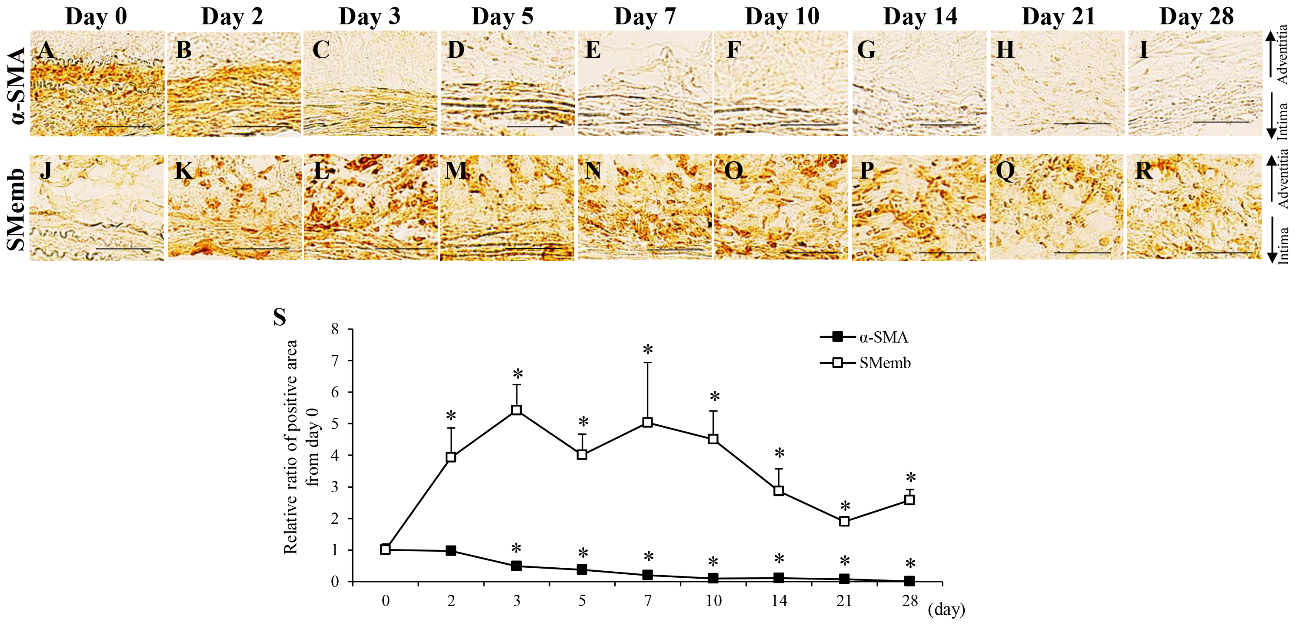


**Figure S8:** Time-dependent changes of contractile vascular smooth muscle (α-smooth muscle actin (α-SMA)) and synthetic vascular smooth muscle (SMemb) from day 0 to 28. (**A**-**I**) Representative images of the immunostaining for α-SMA (scale bar = 50 µm). (**J**-**R**) Representative images of the immunostaining for SMemb (scale bar = 50 µm). (**S**) Quantification of the relative ratio from day 0 of areas positive for α-SMA and SMemb in the vascular wall. Data are expressed as the mean ± SEM. **P* < 0.05 versus day 0. α-SMA: day 0 (n = 7), day 2 (n = 6), day 3 (n = 7), day 5 (n = 7), day 7 (n = 5), day 10 (n = 6), day 14 (n = 6), day 21 (n = 12) and day 28 (n = 5), and SMemb: day 0 (n = 7), day 2 (n = 6), day 3 (n = 8), day 5 (n = 7), day 7 (n = 6), day 10 (n = 6), day 14 (n = 6), day 21 (n = 10) and day 28 (n = 6).


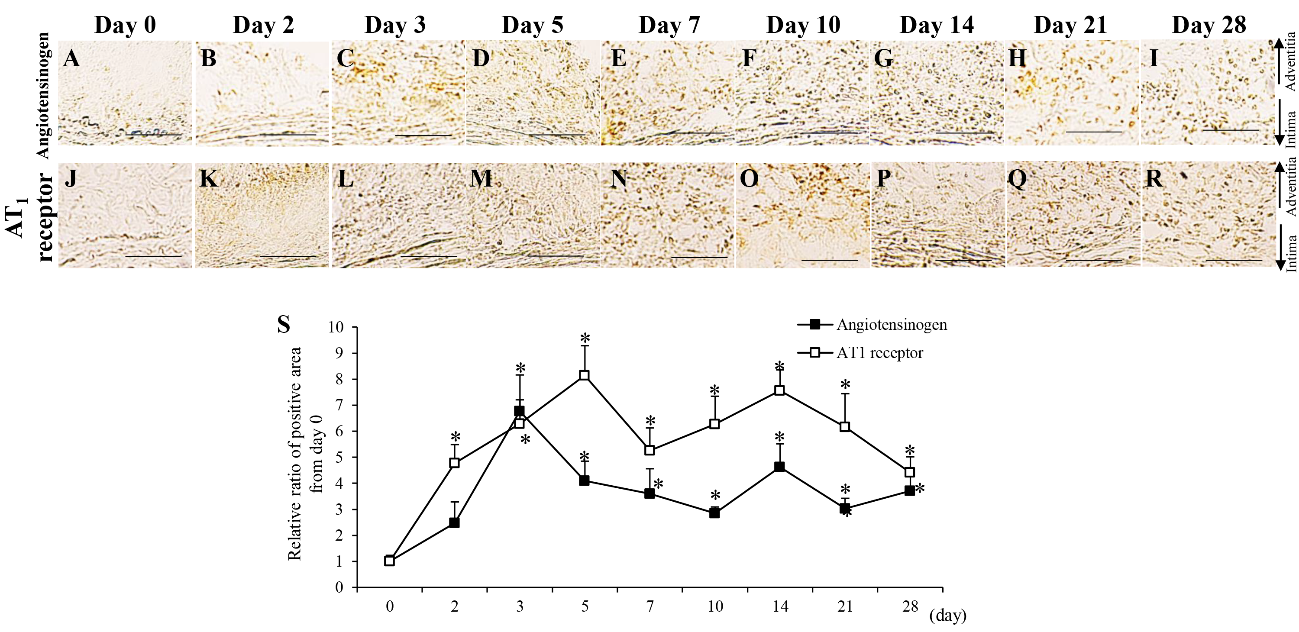


**Figure S9:** Time-dependent changes of angiotensinogen, and angiotensin II type 1 (AT_1_) receptor from day 0 to 28. (**A**-**I**) Representative images of the immunostaining for angiotensinogen (scale bar = 50 µm). (**J**-**R**) Representative images of the immunostaining for AT_1_ receptor (scale bar = 50 µm). (**S**) Quantification of the relative ratio from day 0 of areas positive for angiotensinogen and AT_1_ receptor in the vascular wall. Data are expressed as the mean ± SEM. **P* < 0.05 versus day 0. angiotensinogen: day 0 (n = 7), day 2 (n = 5), day 3 (n = 7), day 5 (n = 7), day 7 (n = 5), day 10 (n = 6), day 14 (n = 6), day 21 (n = 9) and day 28 (n = 5), and AT_1_ receptor: day 0 (n = 7), day 2 (n = 5), day 3 (n = 8), day 5 (n = 7), day 7 (n = 7), day 10 (n = 6), day 14 (n = 6), day 21 (n = 9) and day 28 (n = 6).


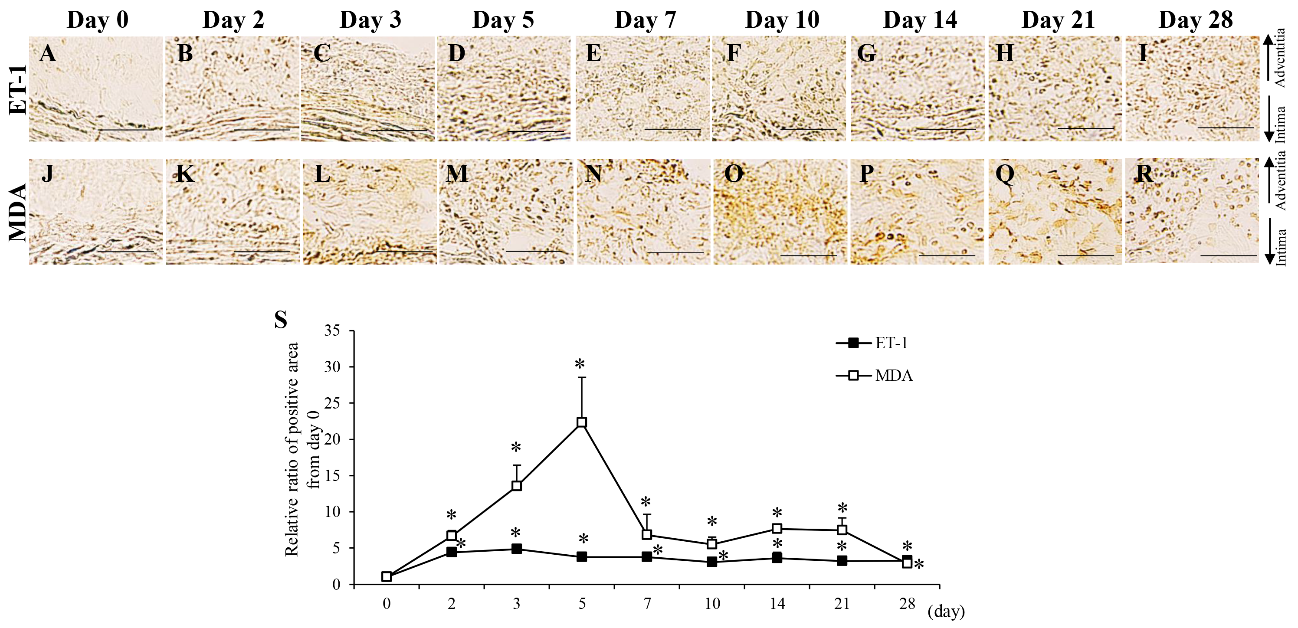


**Figure S10:** Time-dependent changes of endothelin-1 (ET-1) and malondialdehyde (MDA) from day 0 to 28. (**A**-**I**) Representative images of the immunostaining for ET-1 (scale bar = 50 µm). (**J**-**R**) Representative images of the immunostaining for MDA (scale bar = 50 µm). (**S**) Quantification of the relative ratio from day 0 of areas positive for ET-1 and MDA receptor in the vascular wall. Data are expressed as the mean ± SEM. **P* < 0.05 versus day 0. ET-1: day 0 (n = 7), day 2 (n = 6), day 3 (n = 8), day 5 (n = 7), day 7 (n = 6), day 10 (n = 6), day 14 (n = 6), day 21 (n = 9) and day 28 (n = 6), and MDA: day 0 (n = 6), day 2 (n = 6), day 3 (n = 6), day 5 (n = 7), day 7 (n = 6), day 10 (n = 6), day 14 (n = 5), day 21 (n = 5) and day 28 (n = 7).


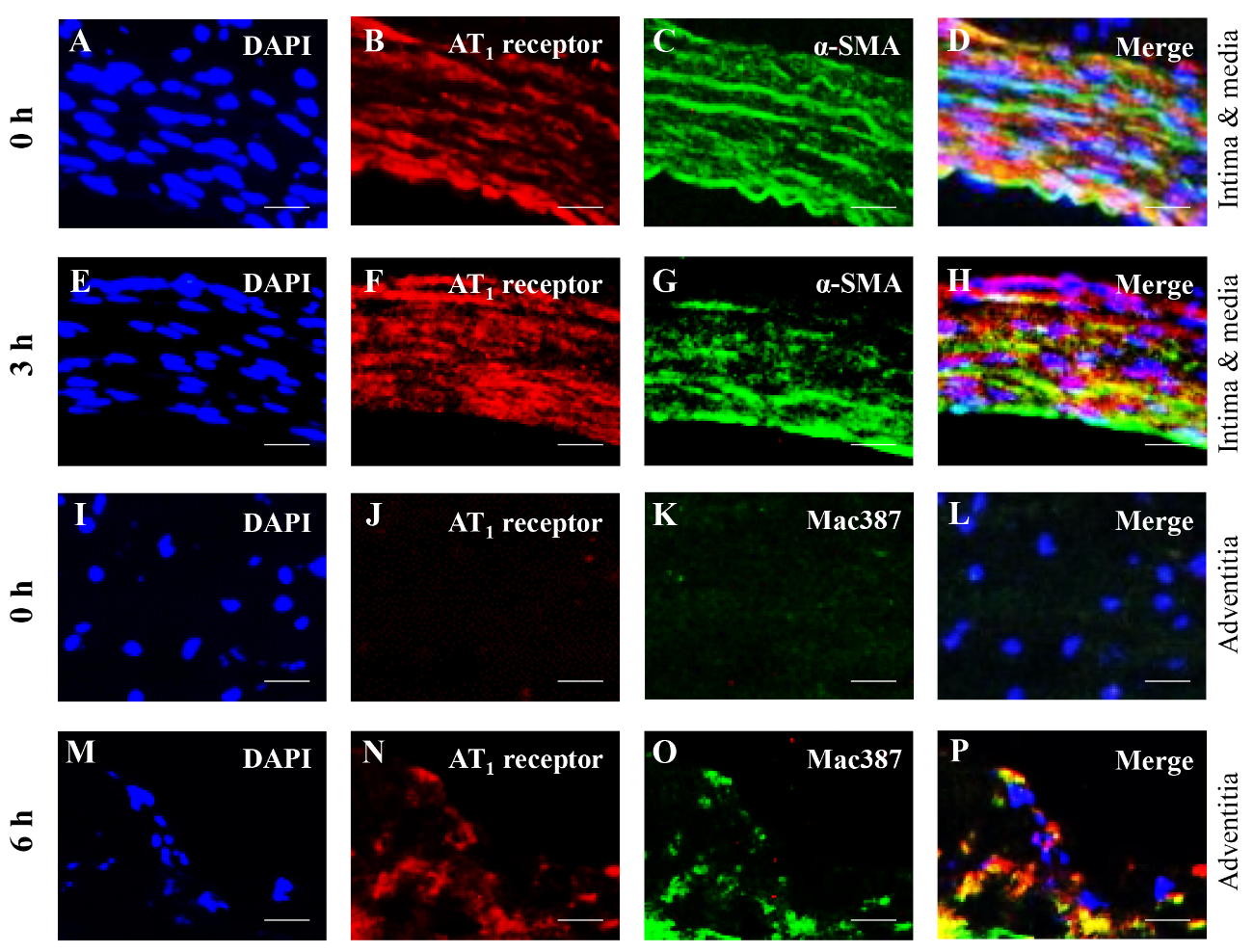


**Figure S11:** Localization of angiotensin II type 1 (AT_1_) receptor, α-smooth muscle actin (α-SMA) ^+^ cells and mac387^+^ macrophages in the aortic wall. Double-immunostaining for AT_1_ receptor and α-SMA in 0 hour (**A-D**) and 3 hours (**E-H**). Double-immunostaining for AT_1_ receptor and mac387^+^ macrophages in 0 hour (**I-L**) and 6 hours (**M-P**). Scale bar = 30 µm. 0 h (n = 5), 3 h (n = 5), and 6 h (n = 5).


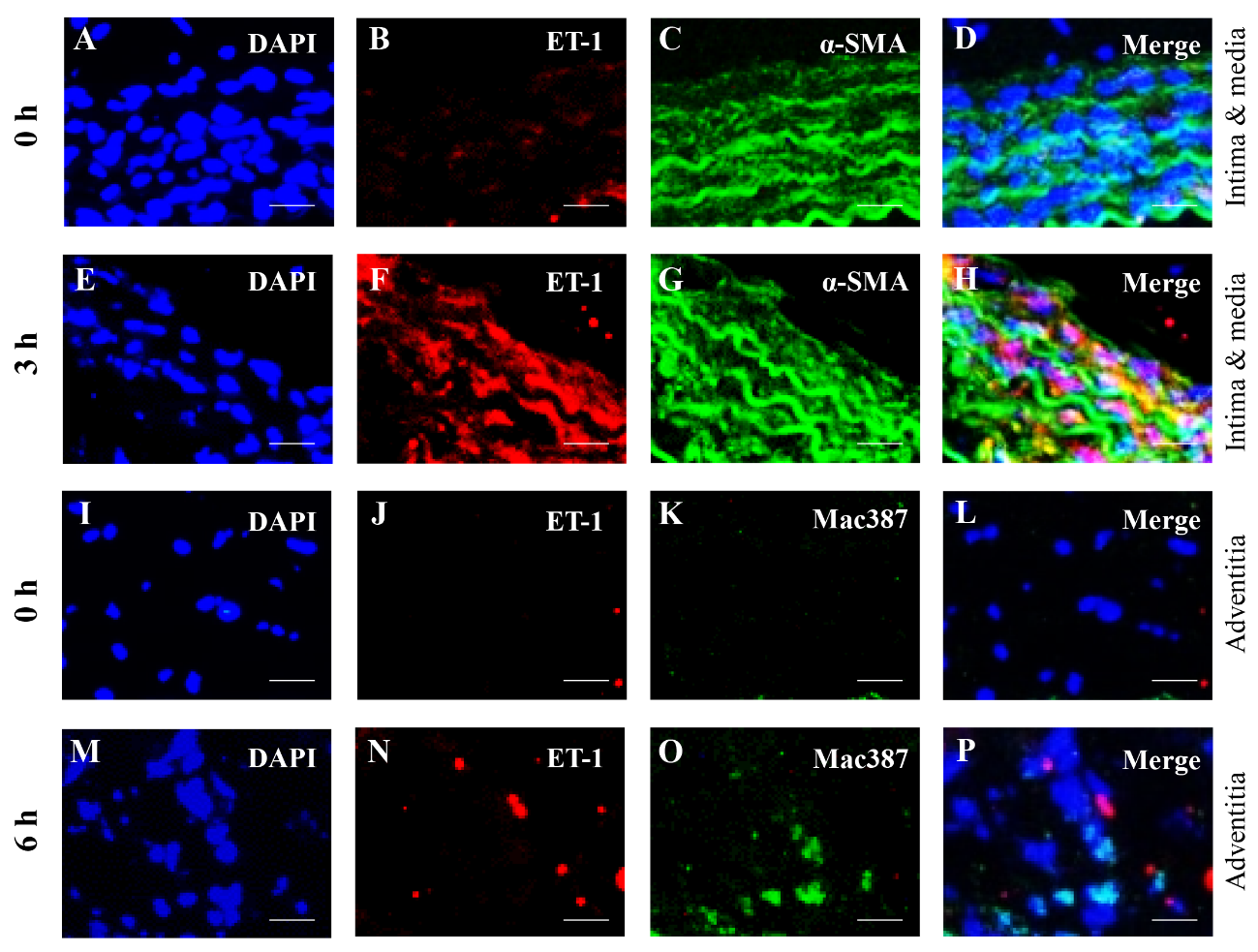


**Figure S12:** Localization of endothelin-1 (ET-1), α-smooth muscle actin (α-SMA) ^+^ cells and mac387^+^ macrophages in the aortic wall. Double-immunostaining for ET-1 and α-SMA in 0 hour (**A-D**) and 3 hours (**E-H**). Double-immunostaining for ET-1 and mac387^+^ macrophages in 0 hour (**I-L**) and 6 hours (**M-P**). Scale bar = 30 µm. 0 h (n = 5), 3 h (n = 5), and 6 h (n = 5).


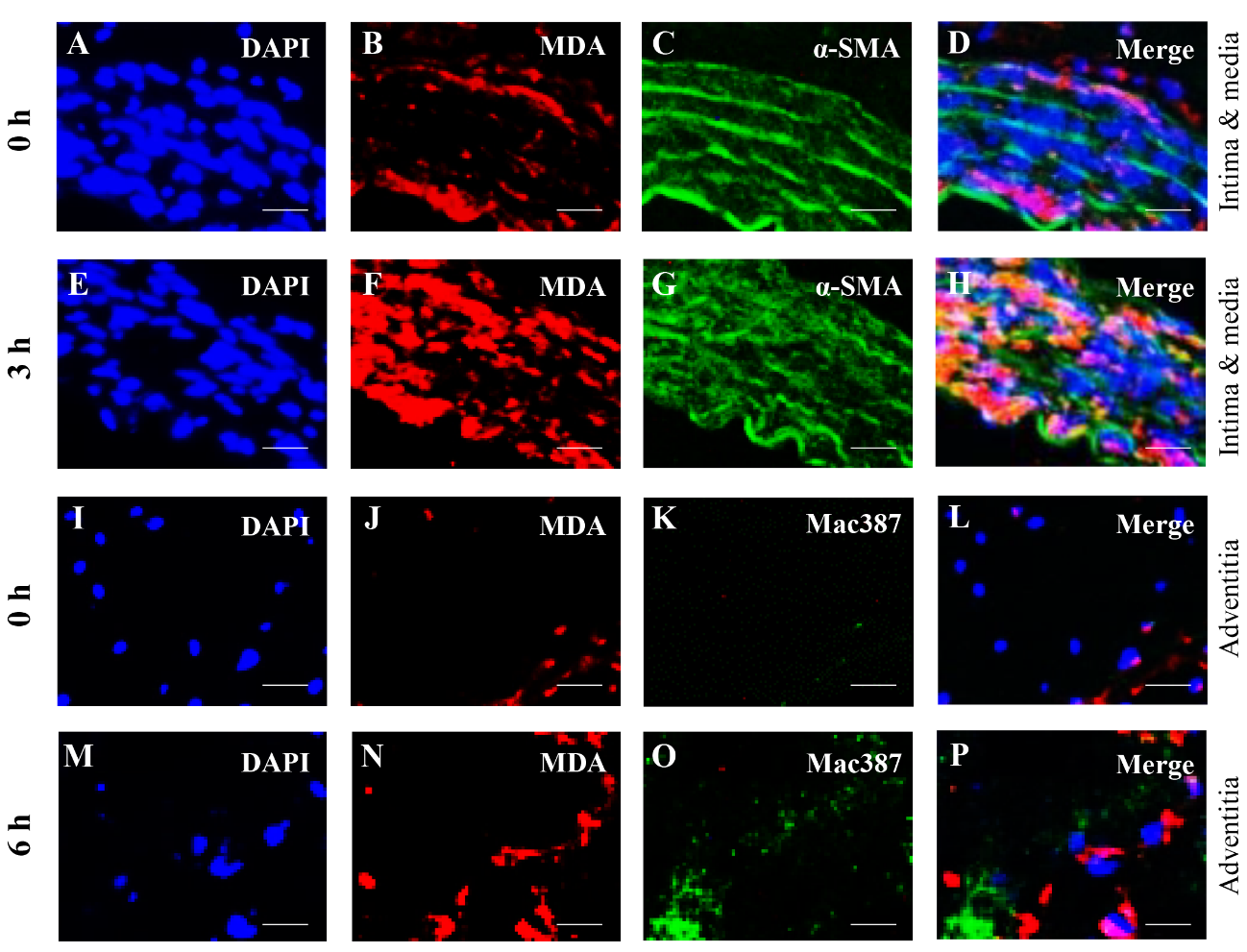


**Figure S13:** Localization of malondialdehyde (MDA), α-smooth muscle actin (α-SMA) ^+^ cells and mac387^+^ macrophages in the aortic wall. Double-immunostaining for MDA and α-SMA in 0 hour (**A-D**) and 3 hours (**E-H**). Double-immunostaining for MDA and mac387^+^ macrophages in 0 hour (**I-L**) and 6 hours (**M-P**). Scale bar = 30 µm. 0 h (n = 5), 3 h (n = 5), and 6 h (n = 5).


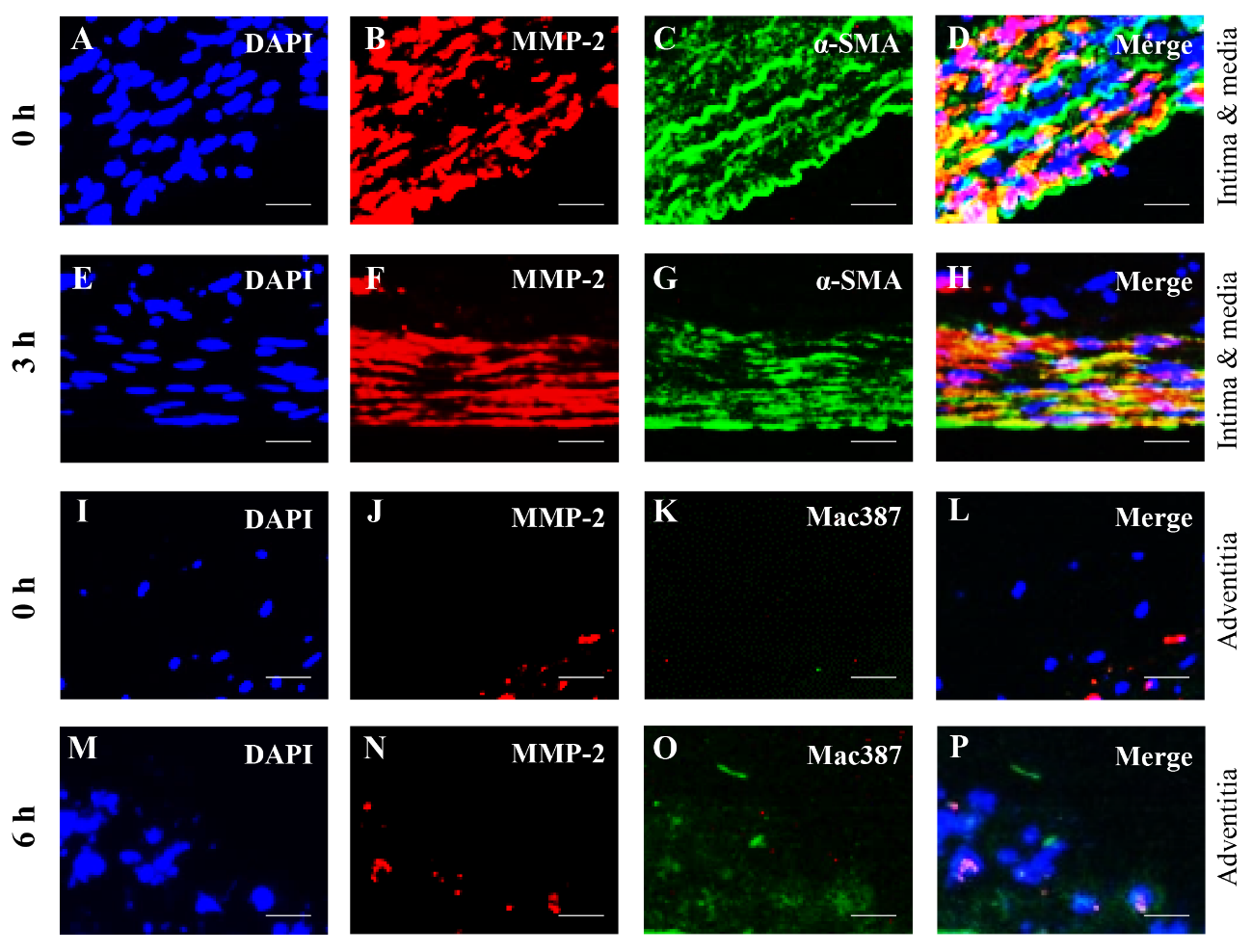


**Figure S14:** Localization of matrix metalloproteinase (MMP) -2, α-smooth muscle actin (α-SMA) ^+^ cells and mac387^+^ macrophages in the aortic wall. Double-immunostaining for MMP-2 and α-SMA in 0 hour (**A-D**) and 3 hours (**E-H**). Double-immunostaining for MMP-2 and mac387^+^ macrophages in 0 hour (**I-L**) and 6 hours (**M-P**). Scale bar = 30 µm. 0 h (n = 5), 3 h (n = 5), and 6 h (n = 5).


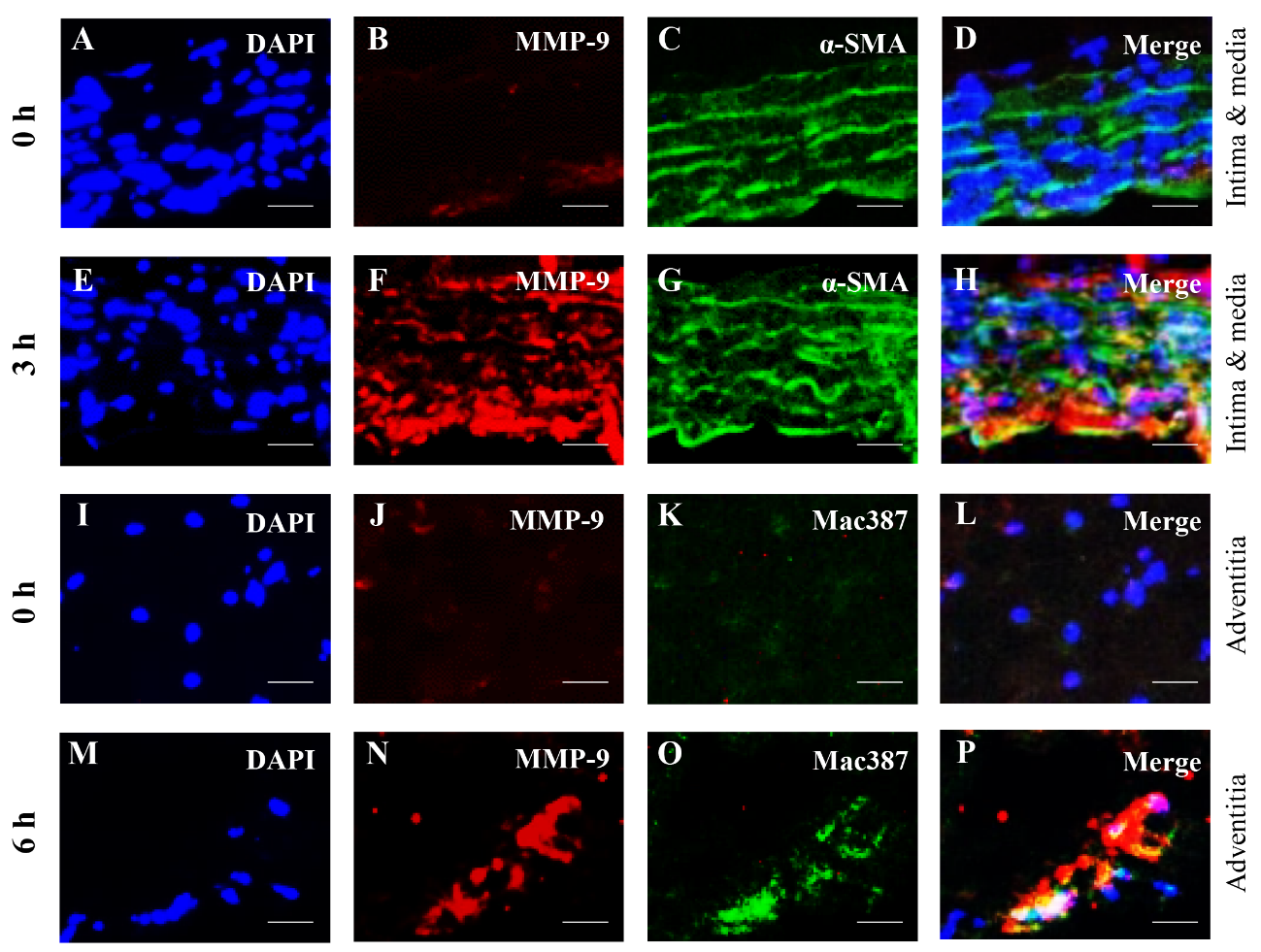


**Figure S15:** Localization of matrix metalloproteinase (MMP) -9, α-smooth muscle actin (α-SMA) ^+^ cells and mac387^+^ macrophages in the aortic wall. Double-immunostaining for MMP-9 and α-SMA in 0 hour (**A-D**) and 3 hours (**E-H**). Double-immunostaining for MMP-9 and mac387^+^ macrophages in 0 hour (**I-L**) and 6 hours (**M-P**). Scale bar = 30 µm. 0 h (n = 5), 3 h (n = 5), and 6 h (n = 5).


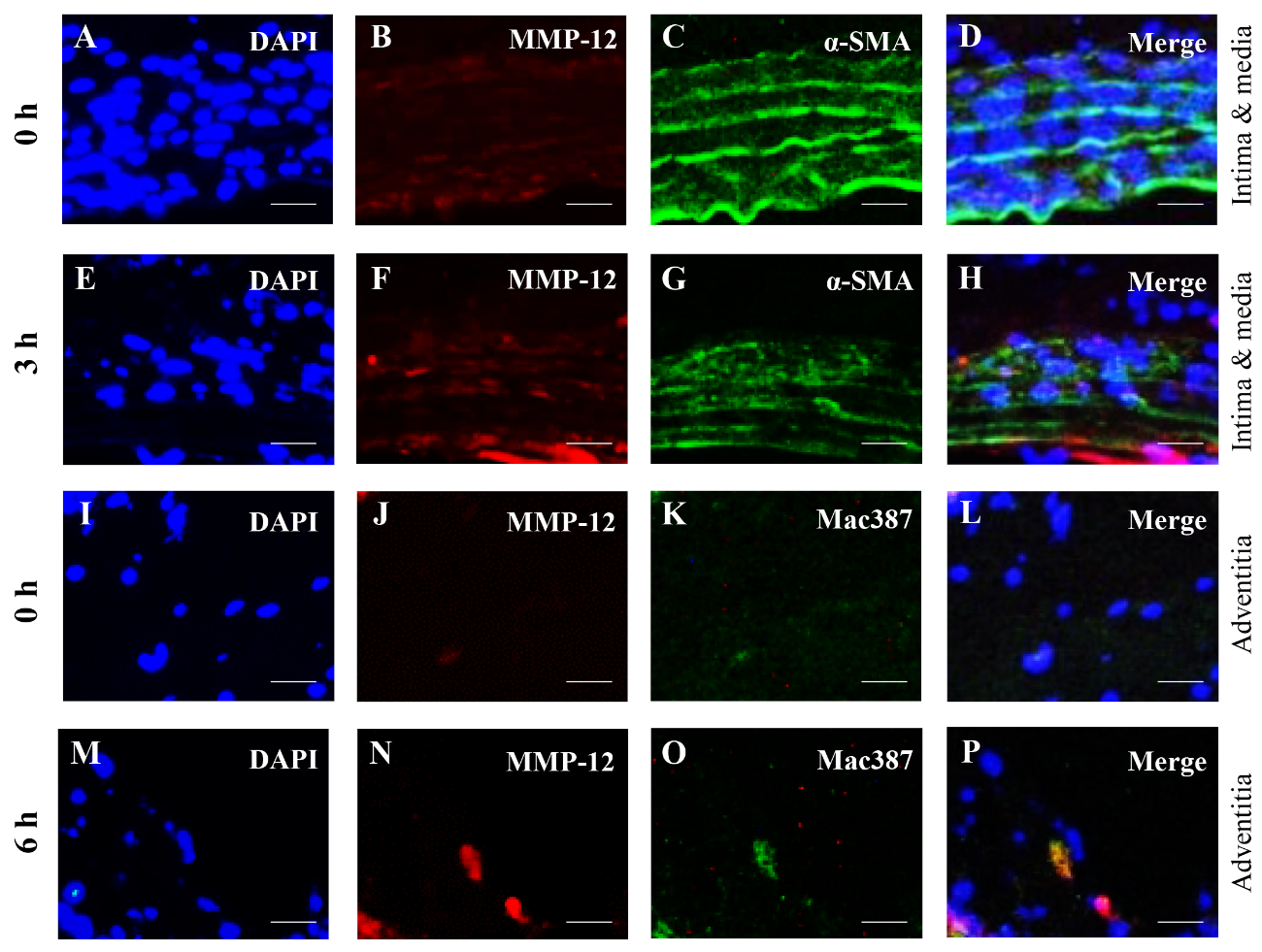


**Figure S16:** Localization of matrix metalloproteinase (MMP) -12, α-smooth muscle actin (α-SMA) ^+^ cells and mac387^+^ macrophages in the aortic wall. Double-immunostaining for MMP-12 and α-SMA in 0 hour (**A-D**) and 3 hours (**E-H**). Double-immunostaining for MMP-12 and mac387^+^ macrophages in 0 hour (**I-L**) and 6 hours (**M-P**). Scale bar = 30 µm. 0 h (n = 5), 3 h (n = 5), and 6 h (n = 5).


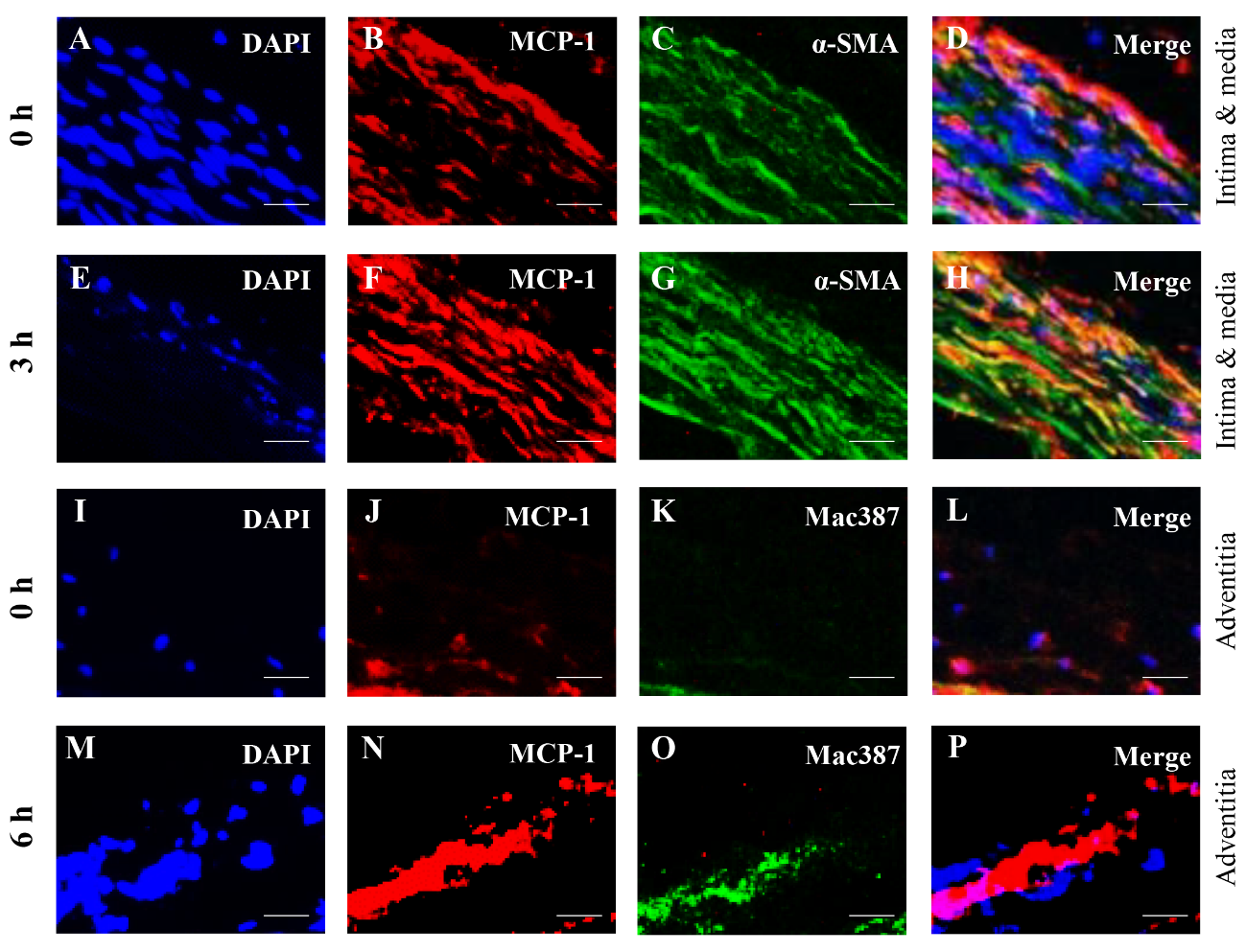


**Figure S17:** Localization of monocyte chemoattractant protein-1 (MCP-1), α-smooth muscle actin (α-SMA) ^+^ cells and mac387^+^ macrophages in the aortic wall. Double-immunostaining for MCP-1 and α-SMA in 0 hour (**A-D**) and 3 hours (**E-H**). Double-immunostaining for MCP-1 and mac387^+^ macrophages in 0 hour (**I-L**) and 6 hours (**M-P**). Scale bar = 30 µm. 0 h (n = 5), 3 h (n = 5), and 6 h (n = 5).


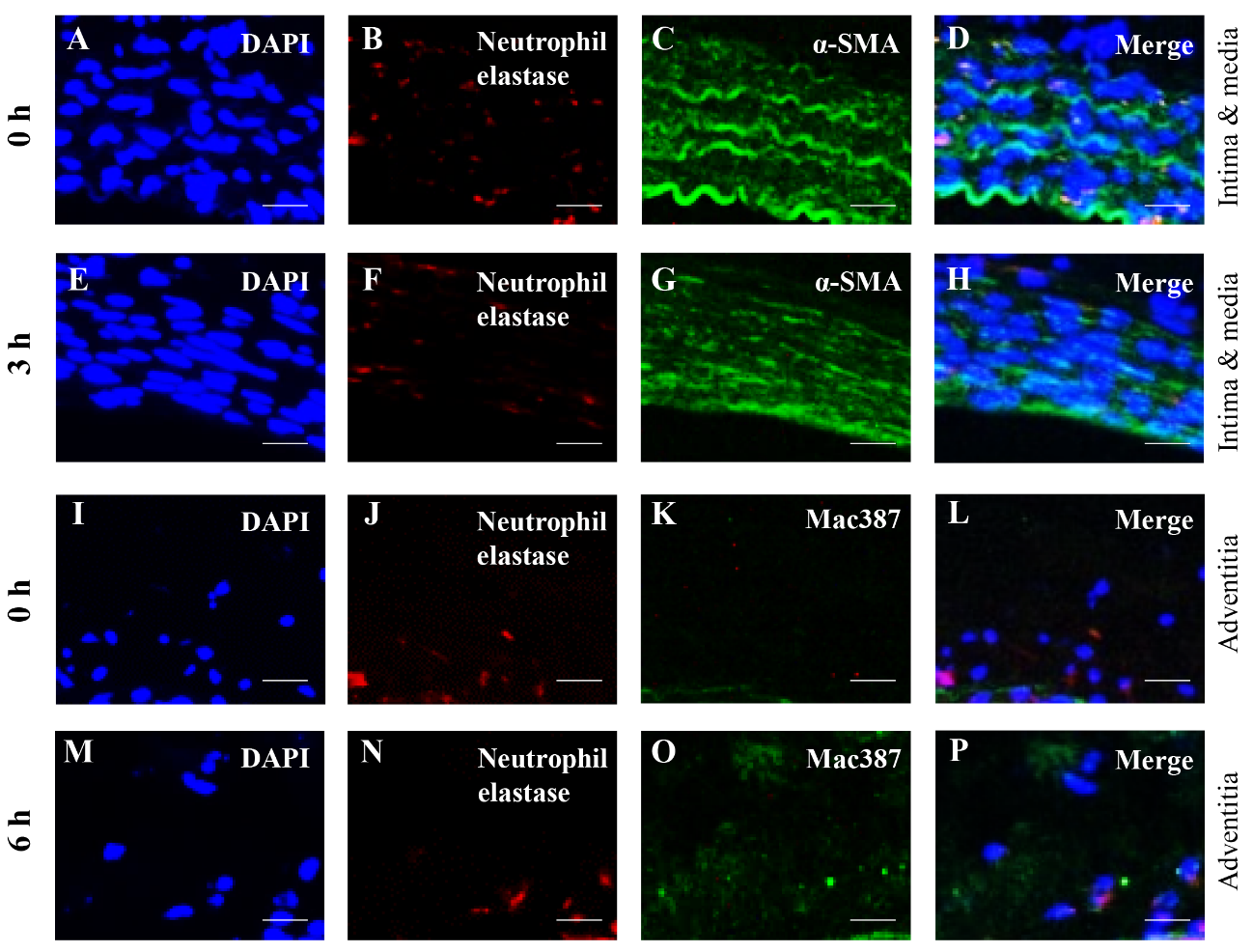


**Figure S18:** Localization of neutrophil elastase, α-smooth muscle actin (α-SMA) ^+^ cells and mac387^+^ macrophages in the aortic wall. Double-immunostaining for neutrophil elastase and α-SMA in 0 hour (**A-D**) and 3 hours (**E-H**). Double-immunostaining for neutrophil elastase and mac387^+^ macrophages in 0 hour (**I-L**) and 6 hours (**M-P**). Scale bar = 30 µm. 0 h (n = 5), 3 h (n = 5), and 6 h (n = 5).


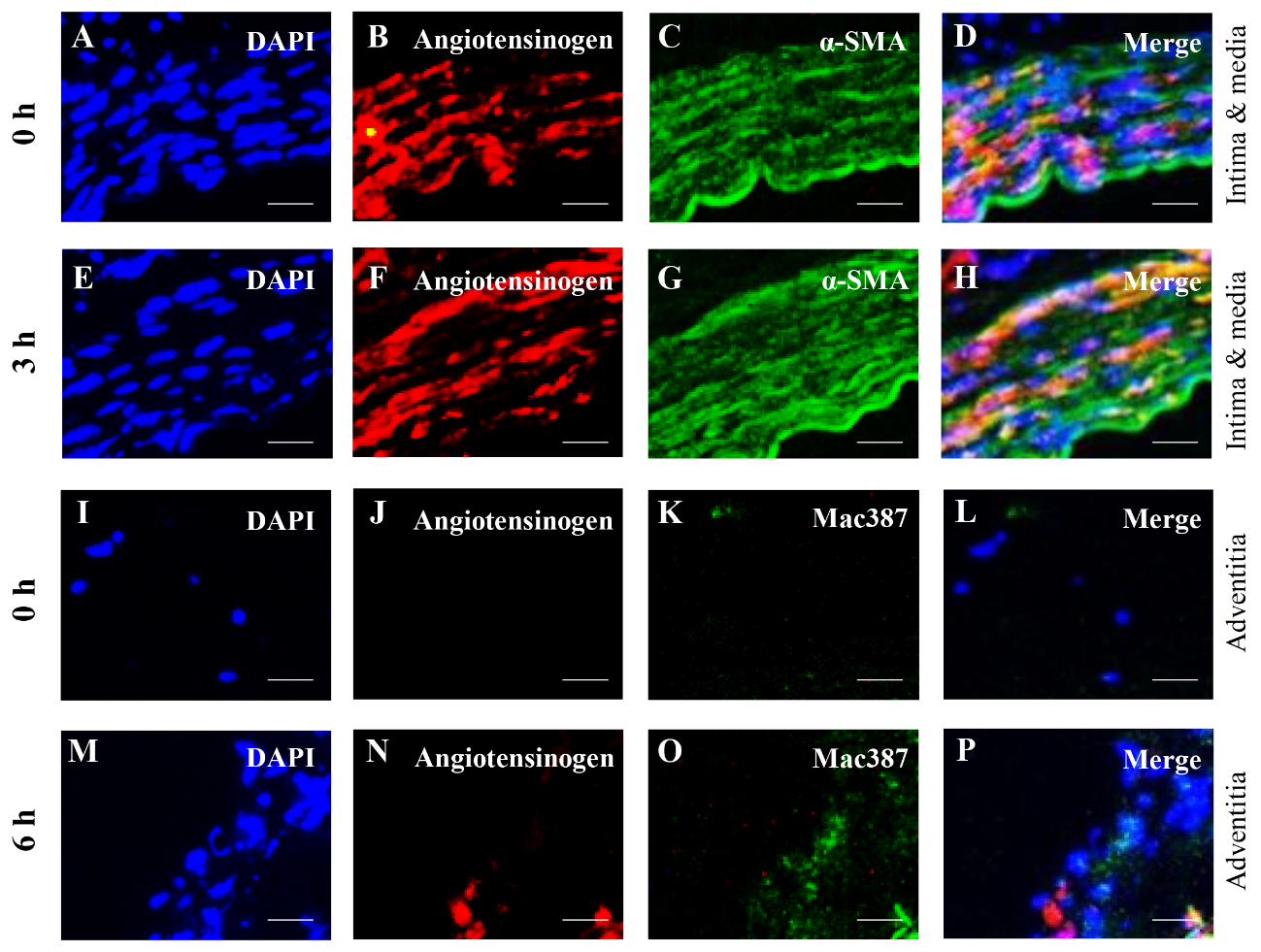


**Figure S19:** Localization of angiotensinogen, α-smooth muscle actin (α-SMA) ^+^ cells and mac387^+^ macrophages in the aortic wall. Double-immunostaining for angiotensinogen and α-SMA in 0 hour (**A-D**) and 3 hours (**E-H**). Double-immunostaining for angiotensinogen and mac387^+^ macrophages in 0 hour (**I-L**) and 6 hours (**M-P**). Scale bar = 30 µm. 0 h (n = 5), 3 h (n = 5), and 6 h (n = 5).
